# Do budget constraints limit access to health care? Evidence from PCI treatments in Hungary

**DOI:** 10.1007/s10754-023-09349-w

**Published:** 2023-04-19

**Authors:** András Kiss, Norbert Kiss, Balázs Váradi

**Affiliations:** 1 KYOS Energy Consulting, Haarlem, The Netherlands; 2 Department of Economics, University of Amsterdam, Amsterdam, The Netherlands; 3 Institute of Management, Corvinus University of Budapest, Budapest, Hungary; 4 Department of Economics, ELTE University, Budapest, Hungary; 5 Budapest Institute for Policy Analysis, Budapest, Hungary

**Keywords:** Hospital funding, Budget cap, Physician agency, Acute myocardial infarction, I110, I180

## Abstract

Under Hungary’s single payer health care system, hospitals face an annual budget cap on most of their diagnoses-related group based reimbursements. In July 2012, percutaneous coronary intervention (PCI) treatments of acute myocardial infarction were exempted from that hospital level budget cap. We use countrywide individual-level patient data from 2009 to 2015 to map the effect of such a quasi-experimental change in monetary incentives on health provider decisions and health outcomes. We find that direct admissions into PCI-capable hospitals increase, especially in central Hungary, where there are several hospitals which can compete for patients. The proportion of PCI treatments at PCI-capable hospitals, however, does not increase, and neither does the number of patient transfers from non-PCI hospitals to PCI-capable ones. We conclude that only patient pathways, plausibly influenced by hospital management, were affected by the shift in incentives, while physicians’ treatment decisions were not. While average length of stay decreased, we do not find any effect on 30-day readmissions or in-hospital mortality.

**Supplementary Information:**

The online version contains supplementary material available at 10.1007/s10754-023-09349-w.

## Introduction

A better understanding of the effects of healthcare reimbursement regimes on provider behavior and health outcomes, and the channels through which they operate, is crucial for evidenced-based policymaking. While a restriction on hospital cost reimbursements beyond an arbitrary threshold can be questioned on ethical grounds, such a budget cap is considered an effective policy tool for cost containment (Moreno-Serra, 2014). There is still only limited evidence, however, about its potential impact on rationing health services or lowering the quality of care.

This study evaluates the effect of a systemwide change in hospital financing targeting the treatment of acute myocardial infarctions (AMI) upon patient pathways, treatment decisions, and health outcomes in Hungary. Hungarian hospitals are reimbursed through a diagnoses-related group (DRG) based system with a cap on the total yearly amount paid to the hospital. In July 2012, percutaneous coronary intervention (PCI) treatments of AMI were exempted from the cap and have been financed fully by the health insurance fund ever since. We use individual-level patient data from 2009 to 2015, covering all AMI cases in the country, to carry out an impact analysis of that regulatory change.

First, we find that the probability that patients get directly admitted to hospitals with PCI capabilities increased right after the financing change from a baseline of 68–76%. The only exceptions were those patients who lived in the immediate proximity of PCI-capable hospitals and already had high direct admission rates. At the same time, transfers from non-PCI hospitals to PCI-capable ones did not substantially decrease, hence the change in regulation did result in more people getting timely access to PCI centers.

Second, the probability of receiving PCI treatment conditional on being admitted to a PCI-capable hospital did not increase after the regulatory change. Overall, this still resulted in more AMI patients receiving PCI treatment, but the effect worked through the patient pathway channel, and not through a change in the decision-making of medical specialists.

Despite the increase in PCI treatments and decrease in average days of hospital stay of AMI patients, we found no convincing evidence of improved outcomes in terms of 30-day readmissions or in-hospital mortality. This null result may be the outcome of our inability to fully control for treatment selection, but it also may be the result of the limited net benefit that the additionally treated patients derive from PCI. Let us note that other, unmeasured dimensions of patient care could have improved, as more AMI patients ended up at larger and better-equipped hospitals.

Our overall conclusion is that applying a hospital-level budget cap does not seem to influence medical decision-making (at least not for potentially life-saving treatments) but can limit hospital admissions. Consequently, lifting the budget cap for expensive emergency care services will likely improve access for those living in a greater distance from specialized centers.

The paper is organized into five sections. The “Related literature” section presents the related literature: since the literature about the impact of budget caps over rationing services or lowering quality of care is scarce, we include further findings about other types of financial pressures (for example, for-profit status) or DRG price changes (also representing a type of financial motivator). The “Background: hospital and AMI treatment financing in Hungary” section provides a background about how hospital services and specifically AMI treatments are financed in Hungary. The “Data and methods” section introduces our datasets and the applied methodology. The “Results” section presents our results, which is followed by a discussion and concluding remarks in the “Discussion and conclusions” section.

## Related literature

The impact of various hospital payment methods on the behavior of hospitals has been analyzed for a long time (see, for example, Langenbrunner & Wiley, 2002). Cost-containment policies have also been spreading in response to growing health care expenditures, although the evaluation of their impact is still an ongoing issue. How and whether the financial constraints that hospital managers and physicians must face under certain provider payment policies affect medical decisions, and therefore treatment costs and quality of care, is a key question for policy making. Budget cap is considered to be an effective policy for cost containment (Moreno-Serra, 2014); however, there is still limited evidence about its potential impact over rationing services or lowering quality of care.

Budget caps can be applied at multiple levels: global (or sector-level) caps limit the total expenditure paid to providers altogether (and thus often use floating or ex-post prices), while hospital-level caps limit expenditure paid to single providers. Sector-level caps involve a cooperation problem among providers: since the marginal revenue of providing an additional unit of service is usually higher than the marginal cost, hospitals are motivated to increase production. This way sector-level caps with floating prices often lead to overproduction. On the other hand, hospital-level budget caps may effectively create a “hard” limit for the volume of services provided, and once the limit is reached, they might discourage hospitals from providing medically justifiable treatments. Consequently, hospital-level budget caps are perceived as much stricter financial constraints by hospitals than sector-level ones.

A Taiwanese study tracing the effects of the introduction of global budgeting found that for-profit and private not-for-profit hospitals, in contrast to government-owned ones, increased treatment intensity among cardiac disease patients, but without improved outcomes (Kan et al., 2014). Higher-tier hospitals and medical centers gained additional patient volume, while local hospitals lost patients as a consequence of global budgeting (Chen & Fan, 2015). The initial introduction of regional budget caps also significantly increased the average claim per AMI patient (due to the cooperation problem inherent in system-wide global budgeting), while the allocation of fixed budget caps to individual hospitals had only a moderate effect (Hsu, 2014). An analysis of incorporating heart stents in the public benefit package in the treatment benefit package in Shanghai, China suggested that the application of a global budget cap results in “provider gaming”: stent usage decreased in the high reimbursement group of AMI patients, where a higher ratio of costs is reimbursed by the third party, also subject to the preset financial ceiling (Yuan et al., 2014).

Maryland’s global budget program set a fixed budgetary limit for each acute care hospital. An evaluation of the first phase pilot did not show reduction in hospital admissions or service volume (Roberts et al., 2018). A later analysis of the full-scale program showed no change in the hospitalization rate for most cardiac patients (except for ischemic stroke admissions) or in 30-day-mortality, but found financial savings among AMI patients, possibly due to reduced 30-day-readmissions (Viganego et al., 2021). A moderate reduction in readmissions without significant changes in spending or mortality was also found among patients undergoing cancer-directed surgery (Offodile et al., 2022).

Hospital financing in the Netherlands mixes several approaches in setting limits. Beyond having a global budget cap with annual expenditure growth targets at sector level, insurers negotiate contracts with individual hospitals; these contracts are typically either set hospital-level global budgets (irrespective of production volume), or contain cost ceilings, where hospitals are reimbursed depending on volume, but only up to a preset level (Gajadien et al., 2022). Hospital-level global budgets (in contrast to cost ceilings) were associated with a lower growth in treatment intensity (measured in spending level), but also with higher probability of at least one hospital visit per year (Gáspár et al., 2020).

While for-profit hospitals treated AMI patients, including the use of interventional cardiac procedures, similarly to non-profit counterparts in a US study (Shah et al., 2007), did not appear to be more responsive to DRG profitability in Taiwan (Liang, 2015), or produced comparable quality to public hospitals in the UK (Moscelli et al., 2018), most studies found that for-profit status or other types of financial pressures tend to have an impact on care quality or treatment decisions. In California, a higher number of uninsured patients and their uncompensated costs resulted in the increase of the mortality rate of insured heart attack patients (Daysal, 2012). Care quality in low-performing hospitals serving patients with lower socio-economic status could be improved by changing financial incentives (Jha et al., 2010). Worse financial position was found to have a moderate impact over patient safety and mortality (Bazzoli et al., 2008). By using quality data of heart attack and heart failure treatments, it was demonstrated that “the lack of financial strength may result in a lower standard of health care services” (Dong, 2015, p. 14). Hospitals having softer budget constraints and less zealous cost control practices appeared to show better mortality outcomes for elderly heart attack patients (Shen & Eggleston, 2009).

Capitation-based payment can also be seen as a tool which puts financial pressure on providers; capitated plans were found to send patients further away to lower-priced hospitals for giving birth, although, there was no evidence of lower quality of care (Ho & Pakes, 2014). Moreover, not only budgetary pressures, but a stronger profit motive can also play a part in medical decisions: private hospitals performed more caesarian sections than public institutions, especially if the cost difference was reimbursed by supplementary health insurance (Milcent & Zbiri, 2022). Patient sorting can also occur as it was found in the case of private providers in the UK (Beckert & Kelly, 2021). Promoting competition in the public system of the UK led to improving clinical outcomes (Gaynor et al., 2013). Recently acquired hospitals seemed to lower their quality regarding patient-experience measures, while no change in clinical indicators was attributed to the transfer of ownership (Beaulieu et al., 2020). Patient satisfaction was found to be negatively associated with increase in hospital concentration and positively with increase in insurance concentration; moreover, the latter was stronger in the case of for-profit hospitals (Hanson et al., 2019).

A few other studies analyzed the impact of price changes in DRG systems upon hospital performance. While our case (lifting the budget cap) did not involve a direct price change, it might be perceived as a similar measure if budget constraints are binding. Increased reimbursement rates in Norway had a positive effect on the volume of medical DRGs (Januleviciute et al., 2016), or on both medical and surgical ones (Melberg et al., 2016). A general price increase in Italy stimulated the number of surgical DRGs, although the adaptation occurred with a time lag of a few years (Verzulli et al., 2017).^1^ It must be noted that price increases might not only lead to increases in quantity or quality of services, but can also trigger upcoding (Shin, 2019). Health systems dominated by public providers can also be prone to upcoding, as demonstrated in, for example, France (Milcent, 2021), Portugal (Barros & Braun, 2017) or Norway (Anthun, 2022; Januleviciute et al., 2016), however, the effect seems to be moderate.

In summary, several studies in the literature suggest that a change in financing conditions can have an impact on treatment decisions. Nonetheless, there remains a lot to learn about the size and channels of the effect. By examining a quasi-experimental financing change targeted at PCI treatments in Hungary, we aim to contribute to filling this research gap.

## Background: hospital and AMI treatment financing in Hungary

The majority of Hungarian hospitals are publicly owned; those which treat AMI patients are all public hospitals (except for one not-for-profit, church-owned PCI center). While public hospitals have, to some degree, always been afflicted by the symptoms of having soft budget constraints (Kornai, 2009; Kornai et al., 2003), such as constantly increasing hospital debts and repeated bailouts by the central government, financial mechanisms certainly play a role in incentivizing hospitals.^2^

Hospital financing in Hungary utilizes a single-payer system where the national health insurance fund directly pays hospitals, and patients can access acute care services free of charge. Physicians are employees of hospitals and typically work for fixed wages. Consequently, financial incentives primarily affect hospital management; while individual medical decisions of physicians are not affected by direct personal financial gains,^3^ they still might be limited by management’s decisions (e.g. concerning rules for accepting patients, availability of resources for treatment options).

Hungary adopted DRG-based payments for reimbursing acute hospital care in 1993. There have been several adjustments to the system over the years (e.g. to DRG codes and weights, or to reimbursement rules of transfer cases and readmissions), but the core elements of DRG-based financing have remained intact. Oversight functions of the national health insurance fund administration body have always been limited, contributing to the appearance of an unintended but predictable consequence, the “DRG creep” (Simborg, 1981). The volume of active hospital cases rose by 36% between 1994 and 2003, while the case-mix-index increased by 12% between 1994 and 1998 (Szummer, 2005). In response to the budgetary pressure, a limit on the sum total of reimbursed DRG weights was introduced in 2004, essentially setting an annual financial ceiling (a budget cap) for each hospital (Endrei et al., 2014).

There were periods when the upper ceiling was fixed and no reimbursement was paid above the cap, and other periods when the ceiling was flexible with partial reimbursement (e.g. 60% reimbursement between 100 and 105% of the cap, but only 10% above 110%). While early research found that hospital managers initially perceived the budget cap as a temporary regulatory tool (Dankó et al., 2006), it has remained remarkably stable over the decades and became an integral part of the Hungarian health care reimbursement system, putting an effective limit on case number and CMI growth. Right before lifting the limit of PCI treatments (the intervention our paper concentrates on) a partial (30%) reimbursement rate over the budget cap was in effect; between July 2011 and June 2012 about 5% of reported DRG volumes fell in this reduced reimbursement category (Endrei et al., 2014, p. 53), indicating that hospitals, in general, were providing treatments over the budget cap. Consequently, policy makers’ expectation that lifting the cap could contribute to an increase in the number of PCI treatments can be considered as reasonable from this perspective. On the other hand, this effect can be mitigated if hospital managements had previously applied preferential treatment towards PCIs in intra-hospital resource allocation (we do not have data about how hospitals internally allocated budget caps to individual departments or medical procedures).

Acute treatments of AMI are generally reimbursed under three DRG codes: one of them stands for AMI without special treatment (e.g. without PCI), while two items of DRG code denote AMI treatment with PCI. Cost-weights of these three DRGs have remained fairly stable during the period under scrutiny (consequently, their financial attractiveness compared to the average hospital case was unchanged), and the incentive change in 2012 did not modify reimbursement fees of AMI treatments. Until June 30, 2012, all three DRG codes were subject to the overall hospital budget cap. From July 1, 2012, PCI treatment codes were removed from the cap and have been financed fully without limit ever since.^4^

## Data and methods

### Data

Our original sample includes all inpatient cases between 2008 and 2015 in Hungary in which the main diagnosis falls in the I21 and I22 ICD-10 categories (acute myocardial infarctions—AMIs). The records are at the level of hospital department, which we aggregate into hospital cases, and contain the age, gender, and ZIP code of the patient, the date of arrival and discharge, as well as diagnoses, treatments, and DRG classification.^5^ We link patient records over the entire 8-year period.

As a further aggregation level, we group consecutive hospital cases into a single AMI episode if a hospital release and a subsequent hospital admission is on the same calendar day. We conduct our empirical enquiries at the episode level.

In our 8-year sample, there are 73 hospitals that regularly treat AMI patients.^6^ 19 hospitals out of the 73 are PCI-capable^7^: 16 hospitals had PCI centers throughout the entire period, and 3 new PCI centers were opened in 2011 and 2013.^8^ All three new centers were placed in countryside hospitals to improve the accessibility of PCI treatments in the southern half of the country. The remaining 54 hospitals are not equipped to perform PCIs, although they still regularly treat AMI patients. We refer to these providers as non-PCI hospitals in the paper.

In order to concentrate on patients with unchanged geographical access conditions to PCI centers, we exclude all patients who live in the catchment areas^9^ of the three newly opened PCI centers, as well as all those patients who were treated by the newly opened PCI centers (wherever they lived). The exclusion helps us avoid confounding the effects of these supply-side shocks.

We also exclude every AMI episode that has a predecessor episode within 1 year (so that our analysis focuses on newly discovered AMI cases), and drop all cases in 2008 for lack of information on predecessor episodes. Moreover, since our event study approach will present year fixed effects before and after the budget cap exemption on PCI treatments, and the exemption entered into force on July 1, 2012, we drop all cases ending before July 1, 2009 or after June 30, 2015.^10^

**Fig. 1:**
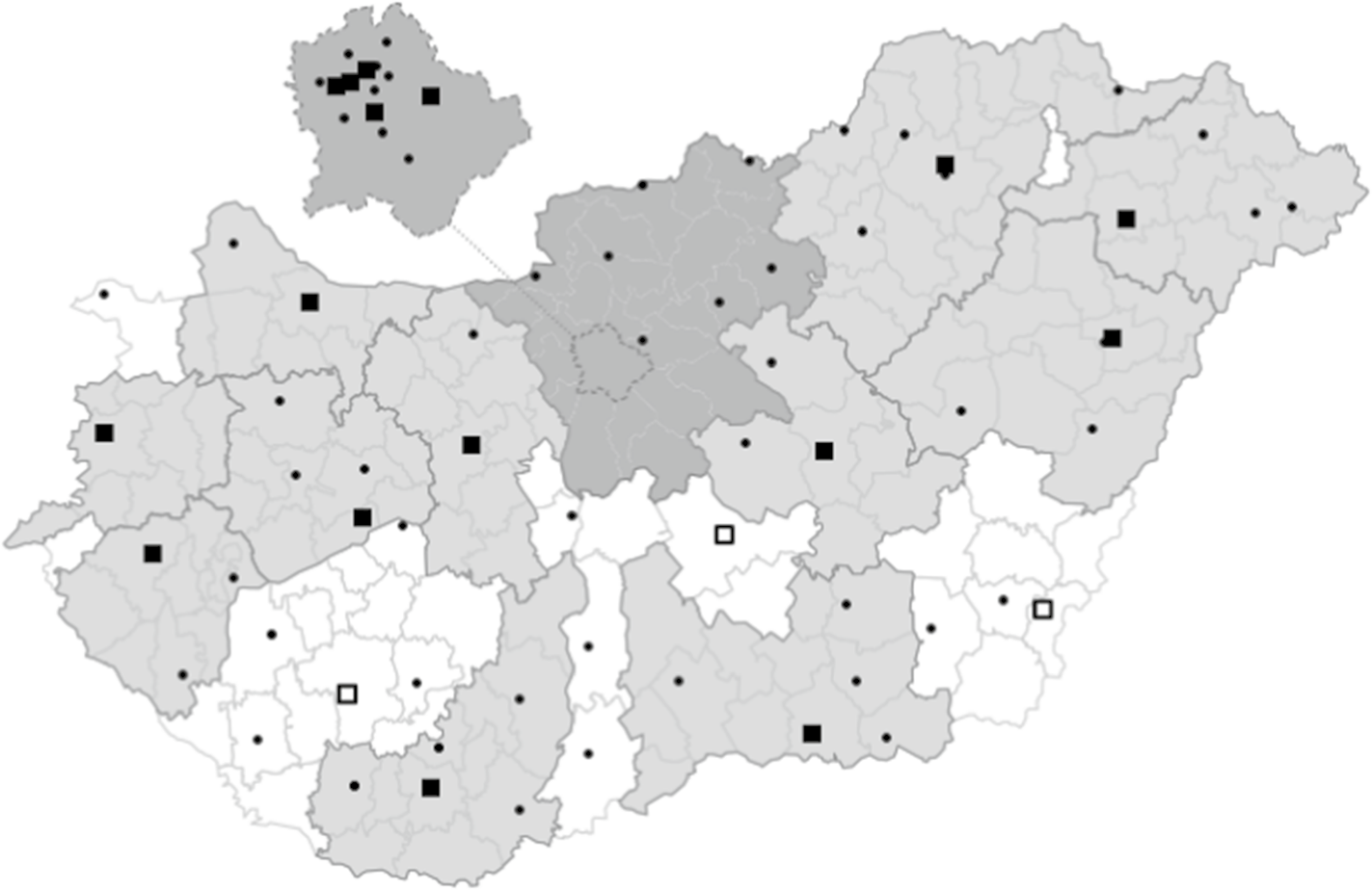
Administrative districts, AMI-treating hospitals, and PCI center catchment areas in central Hungary and the countryside. Note: At the lowest level, the map shows the 175 administrative districts of Hungary. A district is a collection of municipalities (18 on average) with one larger town as its center, forming 10–12% of a county. PCI-capable hospitals are shown as solid black rectangles if they existed throughout our study period, and as white rectangles with a black border if they are newly established. Hospitals that regularly treat AMI patients, but cannot perform PCIs, are shown as black dots. The catchment area of a PCI center consists of those districts (delineated by a thick outer border) in which the majority of patients receive their PCI treatment in the given PCI center. Central Hungary (dark grey background) forms a single catchment area with all 5 of its PCI centers located in Budapest, the capital. Districts with a white background are excluded from the analysis, either because they belong to the catchment area of a newly established PCI center, or because no PCI center performs more than half the PCI treatments in the district. The remaining districts (light grey background) belong to the “countryside” region of our analysis

Since PCI can only be provided in a limited number of hospitals and PCI procedure is time-sensitive, selection of hospital for first admittance matters. There are cases when the patient lives near a non-PCI hospital but the ambulance service can opt for transporting her to a PCI-capable one farther away. In other cases, the nearest hospital is PCI-capable, making it reasonable to admit the patient there even if she does not need a PCI treatment. In Budapest, the capital city of Hungary there are several hospitals with or without PCI, with the differences in access time being small, so that such selection among hospitals can occur more often.^11^

For our main analysis, we have created four geographical subsamples from the main sample based on two factors: (a) accessibility of PCI-capable hospital (whether the nearest AMI hospital has a PCI capability or not); and (b) possibility of choosing a PCI-center (whether there are several hospitals that are considered as relevant choices for first admittance).^12^ Geographic areas are divided into two subgroups using ZIP codes: one subgroup of ZIP codes contains people who are located closer to a PCI-capable than to a non-PCI hospital, while the other subgroup contains people who live closer to non-PCI hospitals. We will refer to these subgroups as *near-PCI* and *near-nonPCI* patients for the rest of the paper.

The four subsamples are separated as follows (see Fig. 1):

- *Central Hungary (near-PCI)* These patients live in the central part of the country, mostly in Budapest, with the nearest hospital being PCI-capable.
- *Central Hungary (near-nonPCI)* These patients live in central Hungary, both inside and outside Budapest, with the nearest hospital not having PCI capabilities.
- *Countryside (near-PCI)* These patients live outside the central Hungary region in municipalities where the nearest hospital has a PCI center. These areas mostly consist of larger cities and their surroundings.
- *Countryside (near-nonPCI)* These patients live in the countryside, typically farther away from large cities, where the nearest hospital does not have PCI capabilities.

### Descriptive statistics

Table 1 presents descriptive statistics for selected variables at the AMI episode level for central Hungary and the countryside separately. Patients are similar in terms of age and gender across the two subsamples, but countryside patients have to travel 50% more on average to reach a PCI-capable hospital. Despite greater distances in the countryside, the lower density of hospitals also means that more people end up in PCI-capable hospitals than in central Hungary (81% vs. 74%).

**Table 1:** Means of selected variables at the AMI episode level in central Hungary and the countryside between July 1, 2009 and June 30, 2015

	Central Hungary	Countryside
Patient age	68.73	67.18
	(0.078)	(0.062)
Share of females	0.422	0.424
	(0.003)	(0.002)
Distance to closest PCI center (mins)	22.08	31.85
	(0.117)	(0.101)
PCI center admission share	0.740	0.812
	(0.003)	(0.002)
Near-PCI patients	0.787	0.953
	(0.006)	(0.002)
Near-nonPCI patients	0.729	0.707
	(0.003)	(0.003)
PCI treatment share	0.576	0.594
	(0.003)	(0.002)
Near-PCI patients	0.587	0.630
	(0.007)	(0.003)
Near-nonPCI patients	0.574	0.568
	(0.003)	(0.003)
Episode length (days)	10.03	10.07
	(0.095)	(0.063)
Hospital cases per episode	1.212	1.151
	(0.003)	(0.002)
30-day readmissions (with AMI)	0.046	0.029
	(0.001)	(0.001)
In-hospital mortality	0.124	0.136
	(0.002)	(0.002)
Observations	28,977	44,639

Looking at relative distance, patients living closer to PCI-capable than to non-PCI hospitals get more frequent admission to PCI centers, even taking hospital transfers into account. The difference is especially marked in the countryside, where 95% of near-PCI patients end up in PCI centers, compared to 70% of near-nonPCI patients. On the other hand, the overall chance of getting PCI treatment is close to equal in central Hungary and the countryside (58–59%); differences between near-PCI and near-nonPCI patients in PCI treatment frequencies are also there, but to a lesser extent.

Patients are transferred between hospitals slightly more often in central Hungary. The average length of AMI episodes, readmission rates and in-hospital mortality patterns are similar.^13^

### Methods

The first methodological issue we face in our impact assessment is that several of our outcome variables of interest show visible time trends in the 3 years before the budget cap exemption.^14^ Thus a simple comparison of before-after means would result in significant differences by virtue of the pre-existing trends.

We deal with this problem by first estimating a univariate regression of each outcome variable on a daily linear trend using the pre-exemption data only. If the estimated pre-trend is significant at the 5% level, we project it onto the post-change years, remove the estimated/projected trend from the entire sample, and use the de-trended outcome variable in the subsequent analysis. If the trend is not significantly different from zero, we use the outcome variable as it is observed. In all of our results below, dependent variables should therefore be understood as de-trended versions of themselves, and treatment effects as measuring changes from what would have happened, had the pre-existing trend continued and the hospital financing system remained unchanged.^15^

**Fig. 2:**
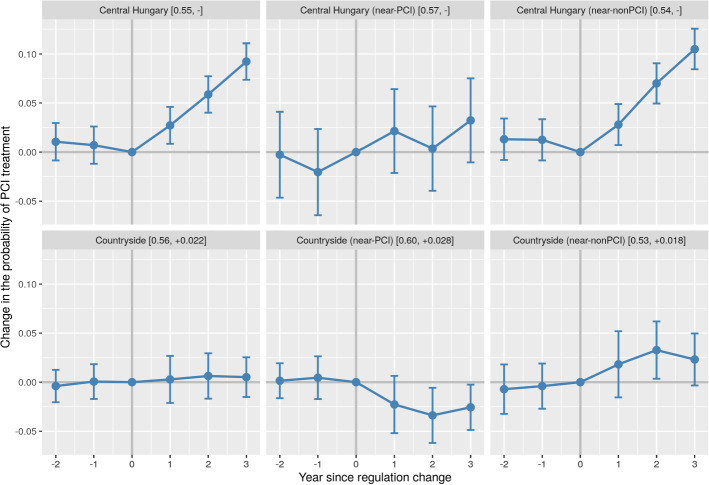
Year fixed effects on PCI treatment probability in different geographical subsamples before and after the budget cap exemption. *Note*: The figure shows the estimated year fixed effects ($\beta$) and 95% confidence intervals on the outcome variable in different geographical samples according to Eq. (1). Pre-treatment years: − 2, − 1, 0 (reference). Post-treatment years: + 1, + 2, + 3. The daily linear trend of the before period—if significantly different from zero at the 5% level—has been removed from the dependent variable prior to the estimation. The brackets in the subfigure headers show the sample-specific unconditional pre-treatment mean of the outcome variable, followed by the removed pre-trend (if any) on a per-year basis. See Table A2 in the online appendix for further details

**Fig. 3:**
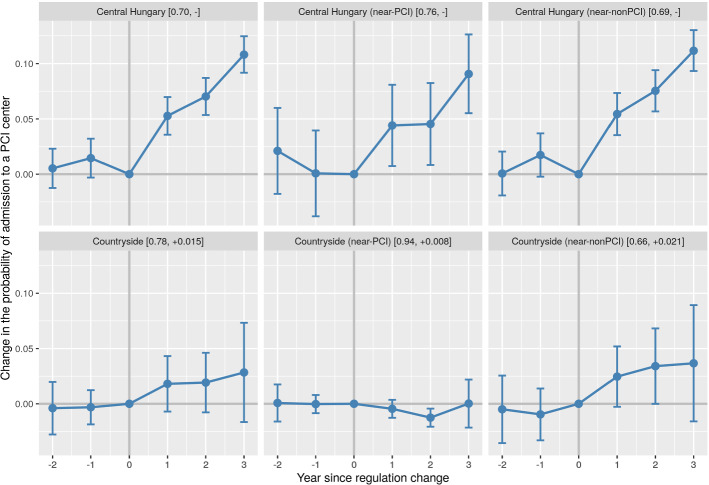
Year fixed effects on overall PCI-capable hospital admission rates in different geographical subsamples before and after the budget cap exemption. Note: The figure shows the estimated year fixed effects ($\beta$) and 95% confidence intervals on the outcome variable in different geographical samples according to Eq. (1). Pre-treatment years: − 2, − 1, 0 (reference). Post-treatment years: + 1, + 2, + 3. The daily linear trend of the before period—if significantly different from zero at the 5% level—has been removed from the dependent variable prior to the estimation. The brackets in the subfigure headers show the sample-specific unconditional pre-treatment mean of the outcome variable, followed by the removed pre-trend (if any) on a per-year basis. See Table A3 in the online appendix for further details

After removing the estimated and projected pre-trend, we run the following linear model for the four geographical subsamples separately:

1 $$\begin{aligned} Y_{i} = \alpha + \beta T_{i} + \gamma B_{i} + \delta X_{i} + u_{i} \end{aligned}$$

where *i* indexes AMI episodes and $Y_{i}$ is an indicator variable for a (de-trended) outcome of interest, such as whether the patient was admitted to a PCI center directly, whether she received PCI treatment, or whether she was readmitted within 30 days with another AMI episode.

$T_{i}$ is our treatment effect variable. In the simplest case, it is an indicator that takes the value of 1 for AMI episodes that fell under the budget cap exemption rule of PCI treatments, and zero otherwise. For our event study regressions, we use year fixed effects relative to the time of regulation change to differentiate between short-term (1 year) and long-term (3 year) changes.^16^

We use two time-based indicator variables $\left( B_{i}\right)$ to capture any potential end-of-period effects that could theoretically arise from budgetary restrictions at the hospital level: (1) last 5 days of each month, and (2) last month of the fiscal year (October) for the hospitals. The performance volume limits are broken down to monthly quotas, hence the inclusion of the last-5-days control. The monthly quotas are non-transferable within the year at the hospital level (although remaining quotas can be used in later months), but they still might be subject to internal negotiation at the department level.^17^

Finally, $X_{i}$ contains gender and age interval^18^ controls, as well as hospital level or hospital catchment area fixed effects^19^ and a dummy variable for weekend admissions. $u_{i}$ is the usual error term.

## Results

We present our results in four stages. First, we describe the evolution of overall PCI treatment probability before and after the budget cap exemption was instituted. In the second and third steps, we investigate potential mechanisms: changes in patient pathways through the health care system and changes in treatment patterns conditional on admission to a PCI-capable hospital. Finally, we look at the effects on three outcome indicators: length of stay, 30-day readmissions with another AMI episode, and in-hospital mortality during the original episode.

We perform each of our analyses on the four geographical subsamples introduced earlier, as well as on the full sample, and present results side-by-side. We first look at a simple binary treatment variable (signifying a period of 3 years before and 3 years after the regulation change). Whenever we find statistically and economically meaningful differences, we further investigate the resulting patterns by substituting the binary treatment variable with year fixed effects. Our criteria for attributing a change in an outcome variable to the change in financing regime are: (1) the effect should show up right after the regime change, and (2) it should not disappear in later years. We also consider causality more plausible whenever the outcome variable shows no pre-trend. Table 2, which we will refer to repeatedly, summarizes our main results with binary treatment. The Online appendix contains details about each of our additional claims.

**Table 2:** Summary of changes in AMI-related health care variables before and after the budget cap exemption of PCI treatments

	Full sample	Central Hungary (near-PCI)	Central Hungary (near-nonPCI)	Countryside (near-PCI)	Countryside (near-nonPCI)
PCI: all patients	0.033***	0.027**	0.059***	− 0.029**	0.028***
	(0.007)	(0.013)	(0.006)	(0.014)	(0.010)
	[0.56, + 0.011]	[0.57, –]	[0.54, –]	[0.60, + 0.028]	[0.53, + 0.018]
PCI center admissions	0.047***	0.053***	0.075***	− 0.006	0.037***
	(0.009)	(0.011)	(0.005)	(0.005)	(0.012)
	[0.75, + 0.007]	[0.76, –]	[0.69, –]	[0.94, + 0.008]	[0.66, + 0.021]
Direct PCI center admissions	0.076***	0.066***	0.087***	− 0.007	0.069***
	(0.012)	(0.012)	(0.006)	(0.005)	(0.015)
	[0.68, –]	[0.69, –]	[0.61, –]	[0.93, + 0.010]	[0.56, + 0.012]
PCI: PCI center patients	− 0.000	− 0.022	0.006	− 0.026	0.010
	(0.011)	(0.018)	(0.015)	(0.017)	(0.011)
	[0.74, + 0.007]	[0.75, –]	[0.79, –]	[0.64, + 0.024]	[0.80, –]
Days of hospital stay	− 0.718***	− 1.584**	− 1.396***	− 0.480	− 0.838***
	(0.114)	(0.773)	(0.155)	(0.296)	(0.240)
	[10.42, –]	[12.44, –]	[9.99, + 0.263]	[10.25, –]	[10.54, –]
30-day readmissions	− 0.000	0.004	0.001	− 0.002	− 0.000
	(0.003)	(0.006)	(0.003)	(0.003)	(0.003)
	[0.04, –]	[0.04, –]	[0.05, –]	[0.02, –]	[0.04, –]
In-hospital mortality	− 0.003	− 0.032***	− 0.014***	0.013**	− 0.017***
	(0.004)	(0.009)	(0.004)	(0.006)	(0.004)
	[0.14, − 0.004]	[0.15, –]	[0.13, –]	[0.14, − 0.009]	[0.15, –]
Num. obs	73,616	5235	23,742	19,061	25,578

Each cell in the table shows the estimated binary treatment effect ($\beta$) on different outcome variables and in different geographical samples according to Eq. (1). The daily linear trend of the before period—if significantly different from zero at the 5% level—has been removed from the dependent variable prior to the estimation. If location-based fixed effects are included in the regression (see Tables A2–A11 in the online appendix for details), the parentheses show standard errors clustered at the level of the fixed effects variable. Otherwise, the parentheses show robust standard errors. The brackets show the unconditional pre-treatment mean of the outcome variables, followed by the removed pre-trend (if any) on a per-year basis. Sample sizes are shown in the bottom row, except for “PCI: PCI center patients”, where Table A6 contains the relevant numbers

### Aggregate effects on PCI treatment probability

Row 1 of Table 2 shows the before-after difference in aggregate PCI treatment shares in the various samples. On a countrywide level, PCIs increase by 3.2 percentage points (pp) relative to a pre-existing trend of + 1.1 pp per year (pp/y) and a baseline of around 56%. The increase is present almost uniformly in the four subsamples, except for near-PCI patients in the countryside, where we observe a slow-down of an otherwise strong pre-trend of + 2.7 pp/y.

The clearest gains can be seen in the subsamples of central Hungary, especially among the near-nonPCI patients, where the pre-trends are absent and the effect size is almost twice as high (5.8 pp/y) as the country average.

Figure 2 shows more details about the temporal structure of the observed changes. Although the three post-treatment years are everywhere jointly nonzero, the individual years are not always so. The increase in PCI treatment among near-nonPCI patients in central Hungary, however, seems robust. To a lesser extent, near-nonPCI patients in the countryside also benefit.

### Admission pathways

Most AMI patients who are admitted to a PCI-capable hospital are admitted either directly, or by transfer from a non-PCI hospital.^20^ Since treatment by PCI is most effective in a short time window following the onset of the AMI episode in the case of STEMI and high-risk NSTEMI patients, people taken directly to a PCI-capable hospital have a higher chance of receiving PCI than those who are only later—or never—transferred.

PCI rates among AMI patients can therefore be increased by admitting more patients to PCI-capable hospitals directly. Admitting more patients is typically made possible by hospital management’s decision, for example, by giving a signal towards ambulance services or primary care providers that they are willing to admit more patients.^21^

A second channel by which overall PCI rates can increase is by transferring a higher share of patients who are initially admitted to non-PCI hospitals to PCI-capable ones. Here, the medical specialist in the first institution is more involved in the decision making, but potential limits on hospital transfers could still involve a higher-level agreement between the sending and the receiving hospitals’ managements.

As Table 2 shows, both the frequency of overall PCI center admissions (Row 2) and of direct PCI center admissions (Row 3) increase markedly (by 4.7–7.6 pp on a countrywide level) relative to the ex-ante period, with the exception of near-PCI countryside patients who are already almost hitting the 100% upper bound on admissions.^22^ The overall and the direct admission estimates are also typically close to each other, which means that the increase in direct admissions does not result in a corresponding decrease in non-PCI to PCI-capable hospital transfers (although a limited amount of substitution is visible).^23^

Figure 3, again, shows more details about the temporal structure of the observed changes for overall PCI-capable hospital admissions. The outcome variable jumps by around 5 pp in central Hungary in the first ex-post year, then continues to increase by another 5 pp in the next 2 years. Again, there is no pre-trend, strengthening the case for causality. Near-nonPCI patients in the countryside also benefit consistently, although the relatively constant year effect estimates become imprecise in later years.

### Treatment decisions conditional on PCI-capable hospital admission

After admission to a hospital with PCI capabilities, the cardiologist on duty decides whether PCI treatment is warranted for a patient. Although the decision is primarily a medical one, the patient-specific benefit of the intervention varies continuously along a scale, rather than being a clear binary choice (see Chandra & Staiger, 2007) for a model-based approach). As a result, there are borderline cases with minimal net benefit of PCI relative to traditional treatment, where secondary considerations, such as the hospital’s financial return to performing PCIs, might swing the balance.^24^ Since the budget cap exemption of PCI treatments generally increases the financial return to performing a PCI, we might expect to see a positive effect on PCI frequency conditional on being admitted to a PCI-capable hospital.

There is, however, a countervailing force as well, which stems from patient selection at the transportation phase. We have shown in the previous section that more AMI patients end up in PCI-capable hospitals after the regulation change. Since pre-hospital patient selection is only based on a subset of PCI appropriateness indicators, the additional patients admitted to PCI-capable hospitals after the budget reimbursement cap was lifted may be less suitable for PCI treatment than the average patient there.^25^ We might, therefore, also expect a slight decline in the frequency of PCI treatment among those patients who were admitted to PCI-capable hospitals.

Row 4 of Table 2 summarizes the evidence on PCI treatment decisions conditional on PCI-capable hospital admission. The majority of the point estimates are mildly negative, but none of them are significant at conventional levels. We would see a similar picture if we conditioned on direct and indirect PCI-capable hospital admissions separately, and also if we looked at more detailed event study graphs. Our results are therefore not inconsistent with the postulates that (1) the additionally admitted PCI center patients after the budget cap exemption are at least somewhat less appropriate for PCI treatment than the average admitted PCI center patient, and (2) medical specialists are not affected by the hospital-level financial incentives provided by the budget cap exemption in their treatment choices.

### Outcomes

We now turn to the analysis of further observable outcomes before and after the regulation change. The short time elapsed since the intervention under scrutiny and the available data only allow us to track length of hospital stay and two early indicators of treatment quality and potential health outcomes: readmissions after an AMI episode and in-hospital mortality during each hospital case. We examine these indicators separately for each geographical group.

#### Length of stay

An average length of stay indicator is often used to measure the efficiency of hospital operations. To arrive at the impact of the policy change, the dependent variable in Eq. (1) is changed to “days of hospital stay”. Row 5 of Table 2 shows a significant decrease of 0.7 days in overall. A significant decrease is also observable in most geographic subsamples: precisely where the probability of patients’ receiving PCI treatment (Row 1) increased. Our conclusion is that the regulation change, and the additional volume of PCI procedures it resulted in, contributed to decreasing average length of hospital stay after an AMI episode.

#### Readmissions

The frequency of 30-day readmissions is a frequently used indicator of AMI treatment quality (Krumholz et al., 2009). Since we are able to track people over time, we can link AMI episodes and mark the ones that are followed within 30 days of a patient’s release by another AMI admission.^26^ In line with our methodology so far, we change the dependent variable in equation (1) to this 30-day AMI readmission indicator.

Row 6 of Table 2 shows the resulting estimates for the different samples. All of the binary treatment coefficients are imprecisely estimated and show no systematic relationship to our PCI center admission and PCI treatment results. We conclude that the behavioral reactions associated with the regulation change have no effect on 30-day AMI readmissions.

#### In-hospital mortality

Corresponding results for an indicator of in-hospital mortality during AMI episodes are shown in Row 7 of Table 2. Although the overall average effect is not significantly different from zero, we do see significant and sizeable effects in each of the four geographic subsamples. Moreover, the direction of the estimates is consistent with the change in PCI treatment probability (Row 1) in each case, although we cannot tell whether it is the treatment or the identity of the hospital that makes the difference,^27^

Despite the encouraging consistency of the binary treatment estimates on in-hospital mortality in Table 2, we are reluctant to conclude that the change in AMI treatment financing has undoubtedly led to better AMI survival chances in Hungary. A visual inspection of the event study graphs in Figure A8 in the online appendix reveals that mortality has been declining before the regulatory change in all of the subsamples where Table 2 shows an improvement. While the ex-ante decline was not marked enough to be picked up as a pre-existing trend at the 5% level, should we have treated it as a trend, its continuation in the post-intervention period would have been sufficient to explain enough of the before-after difference to make the remainder negligible.

## Discussion and conclusions

This paper investigated whether hospital-level budget caps limited the use of PCI for AMI patients before they were relaxed in mid-2012. Our analysis contributes to the literature by evaluating whether budgetary control motivates hospitals to restrict access to potentially life-saving emergency care.

We showed that there were different channels through which the change of financing rules could affect hospital behavior. First, more patients ended up at PCI-capable hospitals after the policy change. The effect was stronger in the region of central Hungary, where several PCI-capable hospitals operate in close proximity to each other (and compete with each other), and even stronger for those who live relatively farther away from these hospitals.

Second, transfers between hospitals did not change, indicating that PCI-capable hospitals are only able to expand their market share if they are able to attract more patients as the first point of inpatient care. Since our case covers an emergency treatment, it is often the ambulance services that decide about the first point of inpatient care. A growing direct admission rate may be the result of better coordination with ambulance services so that they are encouraged to transfer patients directly to PCI centers.

Our patient pathway findings can be interpreted as hospitals expanding their market share among those patients whose treatment became potentially more profitable. Conversely, the financial constraints put on PCI treatments before mid-2012 seem to have played a role in deterring those patients who could more conveniently be treated by nearby non-PCI hospitals.

Furthermore, PCI rates among patients directly or indirectly admitted to hospitals with PCI centers have not increased. This result suggests that the market expansion of PCI-capable hospitals was not well targeted at those patients who truly required PCI, but merely aimed to attract more AMI patients.^28^

Decrease in the average length of hospital stay indicator suggests that the regime change and the corresponding increase in PCI volume led to the possibility of earlier discharges. Finally, outcome indicators such as the 30-day readmission and in-hospital mortality rate did not improve markedly at the introduction of the new financing regime. This does not rule out, however, that other unmeasured dimensions of patient care could have improved as more AMI patients ended up at better-equipped PCI-capable hospitals. Nevertheless, our results are consistent with a recent analysis based on cause-of-death statistics in Hungary: distance from PCI centers was not a strong factor in the territorial heterogeneity of AMI mortality (Uzzoli et al., 2017).

At a higher level of abstraction, our results paint a picture where there is a limit to what can be achieved in acute AMI care by modifying monetary incentives. A simple change in health policy affecting hospital financing: removing a cap on hospital reimbursement did in fact affect certain institutional decisions (observable by way of admission rates) concerning the allocation of AMI patients on the margin, but did not seem to have changed what matters most in terms of health outcomes: *medical* decisions about the treatment of AMI patients have not been influenced by external financial controls. This result can be read in two ways: a celebration of the autonomy of purely medical considerations in the treatment of AMI in Hungarian hospitals on the one hand, and as a cautionary tale about how well-meaning health policy changes using financing incentives may have little effect on what matters most on the other.

A few potential limitations of our study must also be noted. We used administrative data originally produced by hospitals for reimbursement purposes, thus changes in financing rules may have changed how cases are coded, too, for example, budget cap-exempted, fully reimbursed AMI PCI DRGs may become more frequently reported. However, while there might be borderline cases for creative reporting, we did not notice any unexpected increase in case numbers.

Several other factors may also influence our dependent variables in general (e.g. gradual diffusion of knowledge and expertise about PCI treatments) or locally (e.g. changes in medical personnel, management, or internal budgetary customs), that we are unable to measure. To put it in other words: our quantitative analysis treated hospitals as black boxes; peeking inside those black boxes would require additional, possibly qualitative research. Some of these factors, however, are already present in the location-specific fixed effects or the pre-trends, while other factors would require detailed data about hospital, department, or individual level characteristics. Further research could examine why individual hospitals react differently to regulatory changes; however, we must note that the small number of PCI-capable hospitals in the country as well as their relative similarity in responding to financial motivators (all but one are public hospitals) would make such analysis difficult.

Still, our study can inform policy makers about the general impact of budget caps on access to superior treatment options. Our main conclusion is that applying a hospital-level budget cap does not seem to influence medical decision making of physicians, at least, not in the case of a potentially life-saving treatment. On the other hand, it may limit the effective catchment area which a hospital serves: accepting or declining patients from a greater geographic distance seems to be a factor that the hospital management can influence in the Hungarian case. Consequently, lifting the budget cap for expensive emergency care services may improve access of those living in a greater distance from better-equipped specialized centers. But, since marginal cases are affected most, better access does not necessarily lead to improvement in actual health outcomes.

## Supplementary Information

Below is the link to the electronic supplementary material.

Supplementary file1 (PDF 6274 kb)

## Footnotes

1 Price increases might not lead to increases in quantity or quality of services, but can also trigger upcoding (Shin, 2019) Priority setting inside hospitals can also be a very complex process, with several factors at play (Barasa et al., 2014), thus changes in overall financial conditions of hospitals may influence various departments or emergency procedures differently.

2 Until the late 2000s, most hospitals were maintained by local governments. Starting in 2010, ownership was gradually transferred to the central state, with a new supervising agency established in 2012 (Hajnal & Rosta, 2016) Recent qualitative research (Krenyácz, 2017), however, showed that centralization has not resulted in stricter adherence to budgetary limits.

3 At least, not in the area of AMI care, or emergency care in general. Some specialties, like gynecology or elective surgical care, are operated as a mixture of private and public care, and may also be characterized by informal payments, where personal gains of physicians may also come into play.

4 DRG code 2070 stands for AMI without special treatment (e.g. without PCI). Both 2081 and 2082 denote AMI treatment with PCI: 2081 refers to PCI with stents, and 2082 to PCI treatments without stents. However, in practice, the cost of drug-eluting stents (DES) are not reimbursed by using the DRG system, rather, they are directly paid for in a separate channel by the health insurance fund, thus hospitals must report DRG 2082 when they actually use DESs. Since the budget cap was lifted for all PCI treatments, we did not separate PCIs, PCIs with stents, and PCIs with DESs in our analysis. There are some other DRG codes that may be used for AMI patients with temporary pacemakers or thrombolysis but they only represent less than 0.6% of cases; these codes were omitted from analysis. If fully reimbursed, AMI without special treatment resulted in 1189 USD, AMI PCI with stents in 5341 USD, AMI PCI without stents in 3942 USD fee (calculated at a 2012 HUF/USD average exchange rate of 225.37) paid the by the health insurance fund. We do not have reliable cost data about the Hungarian DRGs, but financing experts claim that PCI treatments, when fully reimbursed, positively contribute to financial results of hospitals. Consequently, after the policy change hospitals are financially motivated to attract more patients who require PCI.

5 The ICD version used in Hungarian hospital financing records does not separate ST-elevation (STEMI) and non-ST elevation (NSTEMI) forms of AMI, thus we are unable to perform a separate analysis. The indication and timing of PCI differs in these two cases, with PCI treatment bringing higher net benefits to STEMI patients. On an aggregate level, STEMI cases make up around just under half of all AMI episodes (Jánosi et al., 2017).

6 We applied a cut-off point of at least 15 AMI patients per year on average to classify a hospital as regularly treating AMI patients. There are 30–35 other health care providers in our sample that have fewer than 15 cases per year and are jointly responsible for less than 1% of all yearly cases. They also lack the departments that are typically associated with treating AMIs.

7 We refer to these hospitals either as PCI-capable hospitals, or PCI centers throughout the paper.

8 Investments are not financed by the hospitals themselves from their own revenues but directly from the state budget (or from other development funds). Moreover, the reimbursement of PCI is not automatic either: only those hospitals will be reimbursed which are authorized by the health insurance fund to carry out PCIs. Of course, a hospital can lobby for becoming PCI-capable, but the decision will be based on central capacity planning.

9 A catchment area is defined as a collection of administrative districts in which the majority of PCI treatments are performed by the given PCI center. Central Hungary forms a single catchment area with all 5 of its PCI centers located in Budapest. For a small number of districts in the countryside, no PCI center has a majority in PCI treatments. These are also excluded from the analysis. See Fig. 1 for more details. Apart from the central Hungary/countryside distinction, catchment areas are only used in the analysis to capture the common effects of the PCI centers by calculating standard errors clustered at the catchment area level.

10 As a result, we end up with exactly 3 years of pre-exemption and 3 years of post-exemption data and do not have to complicate the analysis with the inclusion of seasonal effects.

11 The capital is home to 14 hospitals treating AMI patients, and the five largest ones (in terms of AMI case numbers) have PCI centers. These PCI-capable hospitals are geographically concentrated, all being located within a circle of less than 10 miles in diameter, and participating in a joint off-hours and weekend on-call rotation schedule. For almost all patients in Budapest and surrounding central Hungary, any of the five PCI centers in Budapest provide a reasonable alternative, hence separate PCI center catchment areas cannot be delineated. The countryside is set up differently. PCI centers are located in large towns scattered around the rest of the country. In most cases, catchment areas can be clearly separated from one another (often along county borders), and the share of people for whom more than one PCI-capable hospital offers a reasonable alternative is small.

12 Since PCI treatments are profitable for the providers, a limited competition for attracting patients can occur even among public hospitals. They can lobby for more off-hours services, increase their capacity by allocating more staff to avoid the need for redirecting patients, or informally encourage the ambulance service to transport more patients to them.

13 Our measure of in-hospital mortality is at the AMI episode level, so it differs somewhat from the usual hospital-level mortality measures because transfer patients (who survive at least one within-episode hospital stay by definition) only have their last hospital stay of the episode included in the calculation.

14 For example, gradual diffusion of technology, evolving knowledge or continuously improving individual skills of health personnel lead to a slow, year-by-year increase in how often physicians decide to perform PCI.

15 This de-trending procedure allows us to isolate the effect of the regulation change on the outcome variables in a flexible way. Alternatively, we could have estimated pre- and post-trends with a pre-specified trend break, but that approach would have forced more assumptions on our results (e.g. that the effects of the regulation are immediately felt and/or steadily changing over time). We prefer the relative visual richness of the de-trended yearly fixed effects, shown in Figs. 2 and 3.

16 We choose the 12 months preceding the regulation change (from July 1, 2011 until June 30, 2012) as our reference year, relative to which all the other year fixed effect coefficients are estimated.

17 We have no data on how the hospitals in our sample distribute and enforce the overall performance volume limit internally.

18 We group patient age into one of the following seven categories: less than 60 years, 60–85 years in 5-year age groups, and more than 85 years.

19 If the outcome variable refers to transportation patterns (e.g. whether a patient was admitted to a PCI-capable hospital), we use catchment area fixed effects that depend on patient ZIP codes. If the outcome variable refers to treatment decisions conditional on hospital acceptance (e.g. whether a patient who was admitted to a specific PCI-capable hospital received PCI), we use hospital level fixed effects.

20 Typical admission pathways are: (1) single hospital case in PCI-capable or non-PCI hospital; (2) admission to a PCI-capable hospital, then transfer to a non-PCI hospital which is more local to the patient’s residence; (3) admission to a more local non-PCI hospital, then transfer to a PCI center, possibly followed by a re-transfer to the original hospital. More complicated patterns are also observable, but only in a small minority of episodes.

21 Most people with AMI arrive at the hospital by an ambulance car. Choosing the “right” hospital for the patient (PCI-capable or non-PCI one) depends, at least partly, on the ambulance service (which is run separately from the hospitals). However, it is safe to assume that ambulance car personnel are not able to indicate the necessity of PCI as well as cardiologists in hospitals. If the ambulance service is encouraged to directly transport more patients to PCI-capable hospitals, they can most probably justify doing so, even if their guidelines remain unchanged.

22 The recorded ZIP code in our data refers to the patients’ officially registered residence, which may be different from where they are at the moment of their heart attack in a non-trivial number of cases (e.g. due to commuting, weekend or vacation travel, or out-of-date residence information).

23 See Table A5 and Figure A2 in the Online appendix for more evidence concerning changes in hospital transfer case numbers.

24 In addition to patient characteristics, (Chandra & Staiger, 2007) propose that hospital specialization in more or less invasive AMI treatment methods also plays a role in selecting the most advantageous treatment. In particular, they show that the relative benefit of the two treatments is small enough for many people such that they would always benefit more from the one that their hospital is specialized in. Potential substitution for coronary bypass surgery (CABG) can also be examined: our database showed a low number of CABGs without any visible trend over time.

25 For example, the guidelines clearly specify that patients with STEMI, a condition that ambulance personnel can check for, must be transported to a PCI center.

26 Recall that in our main analysis, we do not include AMI episodes that have a predecessor episode within 365 days, thus the episodes that are marked as *being* 30-day readmissions themselves are also not part of the regression samples. Hence we do not track multi-layered readmission frequencies (readmissions of readmissions).

27 In contrast to the specialization argument put forth by Chandra and Staiger (2007) PCI-capable hospitals in Hungary are larger ones and considered to be generally better equipped to treat AMI patients than non-PCI hospitals, regardless of the appropriateness of PCI.

28 Such targeting may not even be possible due to informational constraints at the point of direct admission decisions.
