# Supplementary material for: Do budget constraints limit access to health care? Evidence from PCI treatments in Hungary
Source: Int J Health Econ Manag. 2023 Apr 19;23(2):281–302. doi: 10.1007/s10754-023-09349-w (PMC10156867; doi:10.1007/s10754-023-09349-w)
Supplement: Supplementary file 1 — Supplementary file1 (PDF 6274 kb) [file 10754_2023_9349_MOESM1_ESM.pdf]

A Additional tables and figures (for online publication)

This appendix contains additional tables and figures—some of them referenced in the main text—for online publication.

Table A1 is an extended version of Table 2. It shows the estimated binary treatment effects on all the outcome variables of interest we considered in the analysis.

Tables A2-A11 show detailed regression output corresponding to the econometric models in each cell of Table A1, but with pre- and post-treatment year fixed effects replacing the binary treatment variables of Table A1. For example, Table A2 corresponds to Row 1 of Table A1, Table A3 to Row 2, and so on.

Estimating regression models with pre- and post-treatment year fixed effects also allows us to perform visual event study analyses similar to those in Figure 2 and Figure 3 in the main text. The event study estimates in Figure 2 are shown in more detail in Table A2, and those in Figure 3 are shown in Table A3. The further event study estimates in Tables A4-A11 can be inspected in Figures A1-A8 visually.

Table A1: Summary of changes in AMI-related health care variables before and after the budget cap exemption of PCI treatments

	Full sample	Central Hungary (near-PCI)	Central Hungary (near-nonPCI)	Countryside (near-PCI)	Countryside (near-nonPCI)
PCI: all patients	0.033***	0.027**	0.059***	−0.029**	0.028***
	(0.007)	(0.013)	(0.006)	(0.014)	(0.010)
	[0.56, +0.011]	[0.57, −]	[0.54, −]	[0.60, +0.028]	[0.53, +0.018]
PCI center admissions	0.047***	0.053***	0.075***	−0.006	0.037***
	(0.009)	(0.011)	(0.005)	(0.005)	(0.012)
	[0.75, +0.007]	[0.76, −]	[0.69, −]	[0.94, +0.008]	[0.66, +0.021]
Direct PCI center admissions	0.076***	0.066***	0.087***	−0.007	0.069***
	(0.012)	(0.012)	(0.006)	(0.005)	(0.015)
	[0.68, −]	[0.69, −]	[0.61, −]	[0.93, +0.010]	[0.56, +0.012]
Transfers to PCI centers	0.005	0.025	0.026***	0.012	−0.018
	(0.011)	(0.022)	(0.009)	(0.032)	(0.015)
	[0.21, +0.013]	[0.23, −]	[0.21, −]	[0.18, −]	[0.21, +0.028]
PCI: all PCI center patients	−0.000	−0.022	0.006	−0.026	0.010
	(0.011)	(0.018)	(0.015)	(0.017)	(0.011)
	[0.74, +0.007]	[0.75, −]	[0.79, −]	[0.64, +0.024]	[0.80, −]
PCI: direct PCI center patients	−0.001	−0.032*	0.039**	−0.023	0.011
	(0.012)	(0.017)	(0.015)	(0.018)	(0.011)
	[0.74, +0.008]	[0.75, −]	[0.79, −0.011]	[0.64, +0.023]	[0.81, −]
PCI: transfer PCI center patients	−0.010	0.089***	−0.004	−0.103*	−0.024
	(0.021)	(0.019)	(0.017)	(0.062)	(0.037)
	[0.74, −]	[0.76, −]	[0.75, −]	[0.74, −]	[0.72, −]
Days of hospital stay	−0.718***	−1.584**	−1.396***	−0.480	−0.838***
	(0.114)	(0.773)	(0.155)	(0.296)	(0.240)
	[10.42, −]	[12.44, −]	[9.99, +0.263]	[10.25, −]	[10.54, −]
30-day readmissions	−0.000	0.004	0.001	−0.002	−0.000
	(0.003)	(0.006)	(0.003)	(0.003)	(0.003)
	[0.04, −]	[0.04, −]	[0.05, −]	[0.02, −]	[0.04, −]
In-hospital mortality	−0.003	−0.032***	−0.014***	0.013**	−0.017***
	(0.004)	(0.009)	(0.004)	(0.006)	(0.004)
	[0.14, −0.004]	[0.15, −]	[0.13, −]	[0.14, −0.009]	[0.15, −]
Num. obs.	73,616	5,235	23,742	19,061	25,578

Note: Each cell in the table shows the estimated binary treatment effect (β) on different outcome variables and in different geographical samples according to equation (1). The daily linear trend of the before period—if significantly different from zero at the 5% level—has been removed from the dependent variable prior to the estimation. If location-based fixed effects are included in the regression (see Tables A2-A11 for details), the parentheses show standard errors clustered at the level of the fixed effects variable. Otherwise, the parentheses show robust standard errors. The brackets show the unconditional pre-treatment mean of the outcome variables, followed by the removed pre-trend (if any) on a per-year basis. Sample sizes are shown in the bottom row, except for "Transfers to PCI centers" (Table A5), "PCI: all PCI center patients" (Table A6), "PCI: direct PCI center patients" (Table A7), and "PCI: transfer PCI center patients" (Table A8).

Table A2: Marginal effects on PCI treatment probability among all AMI patients living in a given area

	Full sample	Central Hungary (near-PCI)	Central Hungary (near-nonPCI)	Countryside (near-PCI)	Countryside (near-nonPCI)
Event study estimates (β)					
Year: -2	−0.004	−0.003	0.013	0.001	−0.007
	(0.016)	(0.022)	(0.011)	(0.009)	(0.013)
Year: -1	0.001	−0.020	0.012	0.005	−0.004
	(0.011)	(0.022)	(0.011)	(0.011)	(0.012)
Year: +1	0.015	0.021	0.028***	−0.023	0.018
	(0.010)	(0.022)	(0.011)	(0.015)	(0.017)
Year: +2	0.032***	0.004	0.070***	−0.034**	0.033**
	(0.010)	(0.022)	(0.010)	(0.014)	(0.015)
Year: +3	0.047***	0.032	0.105***	−0.026**	0.023*
	(0.009)	(0.022)	(0.010)	(0.012)	(0.014)
Time of admission controls (γ)					
Weekend admission	0.014**	0.014	0.026***	0.009	0.006
	(0.006)	(0.015)	(0.007)	(0.007)	(0.008)
Last 5 days of the month	−0.002	−0.014	−0.008	0.008	0.000
	(0.003)	(0.017)	(0.008)	(0.010)	(0.007)
Last month of fiscal year	−0.010	0.012	0.001	−0.021	−0.014
	(0.010)	(0.023)	(0.011)	(0.016)	(0.013)
Age-gender controls	Yes	Yes	Yes	Yes	Yes
Referral region fixed effects	Yes	-	-	Yes	Yes
Pre-treatment means	0.56	0.57	0.54	0.60	0.53
Removed pre-trend (y)	0.011	-	-	0.028	0.018
R^2	0.112	0.127	0.105	0.111	0.122
Num. obs.	73,616	5,235	23,742	19,061	25,578

Note: Cells in the upper part of the table show the estimated year fixed effects (β) on PCI treatment probability among all patients in different geographical samples according to equation (1). Pre-treatment years: −2, −1, 0 (reference). Post-treatment years: +1, +2, +3. The projected daily linear trend of the before period—if significantly different from zero at the 5% level—has been removed from the dependent variable prior to the estimation. If location-based fixed effects are included in the regression, the parentheses show standard errors clustered at the level of the fixed effects variable. Otherwise, the parentheses show robust standard errors. The unconditional pre-treatment mean of the outcome variable and the removed pre-trend (if any) on a per-year basis are shown in the bottom panel.

Table A3: Marginal effects on direct or indirect PCI center admissions with AMI

	Full sample	Central Hungary (near-PCI)	Central Hungary (near-nonPCI)	Countryside (near-PCI)	Countryside (near-nonPCI)
Event study estimates (β)					
Year: -2	−0.004	0.021	0.001	0.001	−0.005
	(0.016)	(0.020)	(0.010)	(0.009)	(0.016)
Year: -1	0.002	0.001	0.017*	−0.000	−0.010
	(0.011)	(0.020)	(0.010)	(0.004)	(0.012)
Year: +1	0.033**	0.044**	0.054***	−0.005	0.025*
	(0.015)	(0.019)	(0.010)	(0.004)	(0.014)
Year: +2	0.041**	0.045**	0.075***	−0.013***	0.034*
	(0.016)	(0.019)	(0.010)	(0.004)	(0.017)
Year: +3	0.064***	0.091***	0.112***	0.000	0.037
	(0.018)	(0.018)	(0.009)	(0.011)	(0.027)
Time of admission controls (γ)					
Weekend admission	0.009*	0.037***	0.016**	−0.000	−0.001
	(0.005)	(0.012)	(0.006)	(0.003)	(0.008)
Last 5 days of the month	−0.003	−0.011	−0.008	0.004	0.002
	(0.003)	(0.015)	(0.007)	(0.003)	(0.009)
Last month of fiscal year	−0.000	0.006	0.007	−0.005	−0.003
	(0.005)	(0.019)	(0.010)	(0.005)	(0.008)
Age-gender controls	Yes	Yes	Yes	Yes	Yes
Referral region fixed effects	Yes	-	-	Yes	Yes
Pre-treatment means	0.75	0.76	0.69	0.94	0.66
Removed pre-trend (y)	0.007	-	-	0.008	0.021
R^2	0.120	0.106	0.100	0.086	0.147
Num. obs.	73,616	5,235	23,742	19,061	25,578

Note: Cells in the upper part of the table show the estimated year fixed effects (β) on direct or indirect PCI center admissions in different geographical samples according to equation (1). Pre-treatment years: −2, −1, 0 (reference). Post-treatment years: +1, +2, +3. The projected daily linear trend of the before period—if significantly different from zero at the 5% level—has been removed from the dependent variable prior to the estimation. If location-based fixed effects are included in the regression, the parentheses show standard errors clustered at the level of the fixed effects variable. Otherwise, the parentheses show robust standard errors. The unconditional pre-treatment mean of the outcome variable and the removed pre-trend (if any) on a per-year basis are shown in the bottom panel.

Table A4: Marginal effects on direct PCI center admissions at the onset of an AMI episode

	Full sample	Central Hungary (near-PCI)	Central Hungary (near-nonPCI)	Countryside (near-PCI)	Countryside (near-nonPCI)
Event study estimates (β)					
Year: -2	−0.012	0.022	0.000	0.001	−0.002
	(0.015)	(0.022)	(0.011)	(0.012)	(0.022)
Year: -1	−0.002	0.008	0.008	0.005	−0.009
	(0.011)	(0.022)	(0.011)	(0.004)	(0.019)
Year: +1	0.048***	0.063***	0.059***	−0.003	0.046**
	(0.017)	(0.021)	(0.010)	(0.005)	(0.020)
Year: +2	0.070***	0.059***	0.096***	−0.011**	0.068**
	(0.021)	(0.021)	(0.010)	(0.005)	(0.027)
Year: +3	0.094***	0.105***	0.116***	−0.001	0.081**
	(0.021)	(0.020)	(0.010)	(0.012)	(0.032)
Time of admission controls (γ)					
Weekend admission	0.007	0.027*	0.017**	0.000	−0.007
	(0.006)	(0.014)	(0.007)	(0.003)	(0.009)
Last 5 days of the month	−0.005*	−0.001	−0.011	0.005*	−0.004
	(0.003)	(0.016)	(0.008)	(0.003)	(0.009)
Last month of fiscal year	−0.003	0.001	0.000	−0.001	−0.003
	(0.005)	(0.021)	(0.010)	(0.005)	(0.010)
Age-gender controls	Yes	Yes	Yes	Yes	Yes
Referral region fixed effects	Yes	-	-	Yes	Yes
Pre-treatment means	0.68	0.69	0.61	0.93	0.56
Removed pre-trend (y)	-	-	-	0.010	0.012
R^2	0.118	0.089	0.085	0.088	0.122
Num. obs.	73,616	5,235	23,742	19,061	25,578

Note: Cells in the upper part of the table show the estimated year fixed effects (β) on direct PCI center admissions in different geographical samples according to equation (1). Pre-treatment years: −2, −1, 0 (reference). Post-treatment years: +1, +2, +3. The projected daily linear trend of the before period—if significantly different from zero at the 5% level—has been removed from the dependent variable prior to the estimation. If location-based fixed effects are included in the regression, the parentheses show standard errors clustered at the level of the fixed effects variable. Otherwise, the parentheses show robust standard errors. The unconditional pre-treatment mean of the outcome variable and the removed pre-trend (if any) on a per-year basis are shown in the bottom panel.

Table A5: Marginal effects on transfers to PCI centers following a non-PCI hospital admission with AMI

	Full sample	Central Hungary (near-PCI)	Central Hungary (near-nonPCI)	Countryside (near-PCI)	Countryside (near-nonPCI)
Event study estimates (β)					
Year: -2	−0.003	0.003	0.005	−0.015	−0.011
	(0.024)	(0.035)	(0.014)	(0.021)	(0.011)
Year: -1	0.005	−0.024	0.032**	−0.058	−0.009
	(0.016)	(0.035)	(0.015)	(0.044)	(0.012)
Year: +1	0.005	−0.001	0.030**	−0.016	−0.014
	(0.009)	(0.035)	(0.015)	(0.055)	(0.010)
Year: +2	−0.009	0.005	0.009	−0.038	−0.025
	(0.013)	(0.036)	(0.015)	(0.046)	(0.022)
Year: +3	0.023*	0.060	0.080***	0.031	−0.036*
	(0.012)	(0.039)	(0.016)	(0.055)	(0.018)
Time of admission controls (γ)					
Weekend admission	0.016*	0.060**	0.012	−0.012	0.015
	(0.008)	(0.027)	(0.011)	(0.017)	(0.016)
Last 5 days of the month	0.005	−0.034	0.003	0.009	0.012*
	(0.004)	(0.027)	(0.012)	(0.021)	(0.007)
Last month of fiscal year	0.009	0.022	0.025	−0.068*	0.003
	(0.009)	(0.040)	(0.016)	(0.040)	(0.010)
Age-gender controls	Yes	Yes	Yes	Yes	Yes
Referral region fixed effects	Yes	-	-	Yes	Yes
Pre-treatment means	0.21	0.23	0.21	0.18	0.21
Removed pre-trend (y)	0.013	-	-	-	0.028
R^2	0.069	0.072	0.052	0.115	0.087
Num. obs.	20,599	1,454	8,262	1,081	9,802

Note: Cells in the upper part of the table show the estimated year fixed effects (β) on transfers to PCI centers following a non-PCI hospital admission in different geographical samples according to equation (1). Pre-treatment years: −2, −1, 0 (reference). Post-treatment years: +1, +2, +3. The projected daily linear trend of the before period—if significantly different from zero at the 5% level—has been removed from the dependent variable prior to the estimation. If location-based fixed effects are included in the regression, the parentheses show standard errors clustered at the level of the fixed effects variable. Otherwise, the parentheses show robust standard errors. The unconditional pre-treatment mean of the outcome variable and the removed pre-trend (if any) on a per-year basis are shown in the bottom panel.

Table A6: Marginal effects on PCI treatment probability among AMI patients admitted directly or indirectly to a PCI center

	Full sample	Central Hungary (near-PCI)	Central Hungary (near-nonPCI)	Countryside (near-PCI)	Countryside (near-nonPCI)
Event study estimates (β)					
Year: -2	−0.004	−0.014	0.020	−0.003	−0.015
	(0.015)	(0.034)	(0.027)	(0.014)	(0.014)
Year: -1	−0.003	−0.022	−0.001	0.003	0.001
	(0.007)	(0.015)	(0.011)	(0.010)	(0.013)
Year: +1	−0.011	−0.021	−0.014	−0.022	0.002
	(0.009)	(0.027)	(0.009)	(0.018)	(0.016)
Year: +2	0.001	−0.042	0.019	−0.028*	0.014
	(0.014)	(0.033)	(0.023)	(0.016)	(0.020)
Year: +3	0.001	−0.040*	0.029	−0.029*	0.001
	(0.014)	(0.023)	(0.024)	(0.015)	(0.022)
Time of admission controls (γ)					
Weekend admission	0.005	−0.029	0.012	0.008	0.005
	(0.005)	(0.019)	(0.010)	(0.008)	(0.008)
Last 5 days of the month	−0.000	−0.009	−0.004	0.006	−0.002
	(0.005)	(0.010)	(0.007)	(0.011)	(0.008)
Last month of fiscal year	−0.015	0.008	−0.010	−0.018	−0.019
	(0.009)	(0.029)	(0.008)	(0.017)	(0.015)
Age-gender controls	Yes	Yes	Yes	Yes	Yes
PCI center fixed effects	Yes	Yes	Yes	Y Yes	Yes
Pre-treatment means	0.74	0.75	0.79	0.64	0.80
Removed pre-trend (y)	0.007	-	-	0.024	-
R^2	0.071	0.114	0.044	0.108	0.031
Num. obs.	57,699	4,122	17,318	18,163	18,096

Note: Cells in the upper part of the table show the estimated year fixed effects (β) on PCI treatment probability in different geographical samples according to equation (1). Pre-treatment years: −2, −1, 0 (reference). Post-treatment years: +1, +2, +3. The projected daily linear trend of the before period—if significantly different from zero at the 5% level—has been removed from the dependent variable prior to the estimation. If location-based fixed effects are included in the regression, the parentheses show standard errors clustered at the level of the fixed effects variable. Otherwise, the parentheses show robust standard errors. The unconditional pre-treatment mean of the outcome variable and the removed pre-trend (if any) on a per-year basis are shown in the bottom panel.

Table A7: Marginal effects on PCI treatment probability among patients admitted directly to a PCI center at the onset of an AMI episode

	Full sample	Central Hungary (near-PCI)	Central Hungary (near-nonPCI)	Countryside (near-PCI)	Countryside (near-nonPCI)
Event study estimates (β)					
Year: -2	−0.006	−0.019	0.000	−0.003	−0.023*
	(0.015)	(0.033)	(0.026)	(0.014)	(0.013)
Year: -1	−0.005	−0.027*	−0.012	0.002	−0.004
	(0.007)	(0.016)	(0.011)	(0.010)	(0.012)
Year: +1	−0.014	−0.036*	−0.003	−0.021	−0.004
	(0.010)	(0.020)	(0.010)	(0.019)	(0.014)
Year: +2	−0.001	−0.049	0.040	−0.025	0.010
	(0.015)	(0.033)	(0.026)	(0.017)	(0.018)
Year: +3	−0.001	−0.058***	0.064**	−0.026*	0.001
	(0.014)	(0.019)	(0.028)	(0.015)	(0.018)
Time of admission controls (γ)					
Weekend admission	0.004	−0.033	0.010	0.009	0.006
	(0.005)	(0.021)	(0.009)	(0.008)	(0.007)
Last 5 days of the month	0.002	−0.007	−0.000	0.006	0.001
	(0.005)	(0.007)	(0.007)	(0.010)	(0.010)
Last month of fiscal year	−0.017*	−0.001	−0.015**	−0.019	−0.019
	(0.009)	(0.028)	(0.006)	(0.017)	(0.014)
Age-gender controls	Yes	Yes	Yes	Yes	Yes
PCI center fixed effects	Yes	Yes	Yes	Y Yes	Yes
Pre-treatment means	0.74	0.75	0.79	0.64	0.81
Removed pre-trend (y)	0.008	-	-0.011	0.023	-
R^2	0.080	0.126	0.052	0.109	0.038
Num. obs.	53,017	3,781	15,480	17,980	15,776

Note: Cells in the upper part of the table show the estimated year fixed effects (β) on PCI treatment probability among direct admissions in different geographical samples according to equation (1). Pre-treatment years: −2, −1, 0 (reference). Post-treatment years: +1, +2, +3. The projected daily linear trend of the before period—if significantly different from zero at the 5% level—has been removed from the dependent variable prior to the estimation. If location-based fixed effects are included in the regression, the parentheses show standard errors clustered at the level of the fixed effects variable. Otherwise, the parentheses show robust standard errors. The unconditional pre-treatment mean of the outcome variable and the removed pre-trend (if any) on a per-year basis are shown in the bottom panel.

Table A8: Marginal effects on PCI treatment probability among AMI patients first admitted to a non-PCI hospital, then transferred to a PCI center

	Full sample	Central Hungary (near-PCI)	Central Hungary (near-nonPCI)	Countryside (near-PCI)	Countryside (near-nonPCI)
Event study estimates (β)					
Year: -2	−0.002	0.026	−0.006	−0.094	0.008
	(0.030)	(0.094)	(0.036)	(0.072)	(0.038)
Year: -1	0.004	−0.014	−0.006	0.024	0.010
	(0.019)	(0.054)	(0.011)	(0.078)	(0.038)
Year: +1	−0.012	0.116***	−0.050**	−0.024	0.007
	(0.025)	(0.040)	(0.021)	(0.111)	(0.045)
Year: +2	−0.007	0.016	0.012	−0.270***	−0.011
	(0.030)	(0.055)	(0.028)	(0.087)	(0.054)
Year: +3	−0.009	0.155**	0.016	−0.090	−0.050
	(0.049)	(0.071)	(0.021)	(0.161)	(0.079)
Time of admission controls (γ)					
Weekend admission	0.013	0.018	0.031	−0.098	0.007
	(0.015)	(0.033)	(0.021)	(0.110)	(0.023)
Last 5 days of the month	−0.026	−0.022	−0.034	−0.056	−0.015
	(0.022)	(0.084)	(0.039)	(0.095)	(0.019)
Last month of fiscal year	0.003	0.148***	−0.004	−0.011	−0.014
	(0.025)	(0.049)	(0.032)	(0.147)	(0.025)
Age-gender controls	Yes	Yes	Yes	Yes	Yes
PCI center fixed effects	Yes	Yes	Yes	Y Yes	Yes
Pre-treatment means	0.74	0.76	0.75	0.74	0.72
Removed pre-trend (y)	-	-	-	-	-
R^2	0.034	0.087	0.062	0.165	0.025
Num. obs.	4,682	341	1,838	183	2,320

Note: Cells in the upper part of the table show the estimated year fixed effects (β) on PCI treatment probability among transfer patients in different geographical samples according to equation (1). Pre-treatment years: −2, −1, 0 (reference). Post-treatment years: +1, +2, +3. The projected daily linear trend of the before period—if significantly different from zero at the 5% level—has been removed from the dependent variable prior to the estimation. If location-based fixed effects are included in the regression, the parentheses show standard errors clustered at the level of the fixed effects variable. Otherwise, the parentheses show robust standard errors. The unconditional pre-treatment mean of the outcome variable and the removed pre-trend (if any) on a per-year basis are shown in the bottom panel.

Table A9: Marginal effects on length of hospital stay (in days) during an AMI episode among all AMI patients living in a given area

	Full sample	Central Hungary (near-PCI)	Central Hungary (near-nonPCI)	Countryside (near-PCI)	Countryside (near-nonPCI)
Event study estimates (β)					
Year: -2	−0.061	0.001	0.028	−0.181	0.450*
	(0.206)	(1.116)	(0.278)	(0.300)	(0.256)
Year: -1	−0.096	1.499	−0.114	−0.319	0.076
	(0.194)	(1.977)	(0.291)	(0.281)	(0.289)
Year: +1	−0.656***	−1.023	−0.876***	−0.638*	−0.619**
	(0.171)	(0.987)	(0.289)	(0.385)	(0.301)
Year: +2	−0.574***	0.024	−1.267***	−0.330	−0.724**
	(0.159)	(1.150)	(0.323)	(0.346)	(0.358)
Year: +3	−1.072***	−2.279***	−2.132***	−0.965***	−0.645
	(0.229)	(0.834)	(0.275)	(0.302)	(0.433)
Time of admission controls (γ)					
Weekend admission	−0.566***	−2.579***	−0.610***	−0.171	−0.416***
	(0.115)	(0.599)	(0.156)	(0.173)	(0.107)
Last 5 days of the month	0.343**	0.717	0.092	0.557***	0.334
	(0.147)	(1.231)	(0.216)	(0.172)	(0.240)
Last month of fiscal year	−0.112	0.368	0.018	−0.141	−0.163
	(0.140)	(1.457)	(0.247)	(0.282)	(0.324)
Age-gender controls	Yes	Yes	Yes	Yes	Yes
Referral region fixed effects	Yes	-	-	Yes	Yes
Pre-treatment means	10.42	12.44	9.99	10.25	10.54
Removed pre-trend (y)	-	-	0.263	-	-
R^2	0.014	0.015	0.022	0.017	0.014
Num. obs.	73,616	5,235	23,742	19,061	25,578

Note: Cells in the upper part of the table show the estimated year fixed effects (β) on length of stay in different geographical samples according to equation (1). Pre-treatment years: −2, −1, 0 (reference). Post-treatment years: +1, +2, +3. The projected daily linear trend of the before period—if significantly different from zero at the 5% level—has been removed from the dependent variable prior to the estimation. If location-based fixed effects are included in the regression, the parentheses show standard errors clustered at the level of the fixed effects variable. Otherwise, the parentheses show robust standard errors. The unconditional pre-treatment mean of the outcome variable and the removed pre-trend (if any) on a per-year basis are shown in the bottom panel.

Table A10: Marginal effects on 30-day readmissions with another AMI episode among all AMI patients living in a given area

	Full sample	Central Hungary (near-PCI)	Central Hungary (near-nonPCI)	Countryside (near-PCI)	Countryside (near-nonPCI)
Event study estimates (β)					
Year: -2	−0.003	0.004	−0.009**	−0.000	0.000
	(0.003)	(0.010)	(0.005)	(0.002)	(0.006)
Year: -1	−0.005**	−0.010	−0.007	−0.001	−0.004
	(0.002)	(0.009)	(0.005)	(0.003)	(0.005)
Year: +1	−0.001	0.001	−0.004	−0.003	0.004
	(0.005)	(0.010)	(0.005)	(0.004)	(0.004)
Year: +2	−0.003	−0.000	−0.001	−0.004	−0.004
	(0.003)	(0.010)	(0.005)	(0.003)	(0.004)
Year: +3	−0.004	0.004	−0.009*	−0.001	−0.003
	(0.004)	(0.010)	(0.005)	(0.003)	(0.005)
Time of admission controls (γ)					
Weekend admission	−0.000	0.007	0.002	−0.001	−0.002**
	(0.002)	(0.007)	(0.003)	(0.003)	(0.001)
Last 5 days of the month	0.004**	−0.004	0.007*	0.005**	0.001
	(0.002)	(0.007)	(0.004)	(0.002)	(0.004)
Last month of fiscal year	−0.002	−0.012	0.003	−0.004	−0.003
	(0.003)	(0.009)	(0.005)	(0.004)	(0.004)
Age-gender controls	Yes	Yes	Yes	Yes	Yes
Referral region fixed effects	Yes	-	-	Yes	Yes
Pre-treatment means	0.04	0.04	0.05	0.02	0.04
Removed pre-trend (y)	-	-	-	-	-
R^2	0.006	0.002	0.002	0.003	0.010
Num. obs.	73,616	5,235	23,742	19,061	25,578

Note: Cells in the upper part of the table show the estimated year fixed effects (β) on 30-day readmissions in different geographical samples according to equation (1). Pre-treatment years: −2, −1, 0 (reference). Post-treatment years: +1, +2, +3. The projected daily linear trend of the before period—if significantly different from zero at the 5% level—has been removed from the dependent variable prior to the estimation. If location-based fixed effects are included in the regression, the parentheses show standard errors clustered at the level of the fixed effects variable. Otherwise, the parentheses show robust standard errors. The unconditional pre-treatment mean of the outcome variable and the removed pre-trend (if any) on a per-year basis are shown in the bottom panel.

Table A11: Marginal effects on in-hospital mortality during an AMI episode among all AMI patients living in a given area

	Full sample	Central Hungary (near-PCI)	Central Hungary (near-nonPCI)	Countryside (near-PCI)	Countryside (near-nonPCI)
Event study estimates (β)					
Year: -2	−0.000	−0.002	0.011	−0.001	0.002
	(0.003)	(0.017)	(0.007)	(0.009)	(0.007)
Year: -1	0.003	−0.019	0.009	0.010	0.002
	(0.004)	(0.016)	(0.007)	(0.009)	(0.007)
Year: +1	0.003	−0.023	−0.001	0.029***	−0.012**
	(0.005)	(0.016)	(0.007)	(0.009)	(0.005)
Year: +2	−0.005	−0.044***	−0.008	0.002	−0.012
	(0.004)	(0.016)	(0.007)	(0.010)	(0.009)
Year: +3	−0.005	−0.051***	−0.011	0.018	−0.022***
	(0.005)	(0.015)	(0.007)	(0.011)	(0.006)
Time of admission controls (γ)					
Weekend admission	0.013***	0.008	0.008	0.012	0.019***
	(0.003)	(0.011)	(0.005)	(0.009)	(0.003)
Last 5 days of the month	0.004	0.011	0.003	−0.002	0.006
	(0.003)	(0.012)	(0.006)	(0.007)	(0.007)
Last month of fiscal year	0.004	0.011	0.002	−0.004	0.011
	(0.007)	(0.016)	(0.007)	(0.008)	(0.012)
Age-gender controls	Yes	Yes	Yes	Yes	Yes
Referral region fixed effects	Yes	-	-	Yes	Yes
Pre-treatment means	0.14	0.15	0.13	0.14	0.15
Removed pre-trend (y)	-0.004	-	-	-0.009	-
R^2	0.071	0.088	0.069	0.068	0.074
Num. obs.	73,616	5,235	23,742	19,061	25,578

Note: Cells in the upper part of the table show the estimated year fixed effects (β) on in-hospital mortality in different geographical samples according to equation (1). Pre-treatment years: −2, −1, 0 (reference). Post-treatment years: +1, +2, +3. The projected daily linear trend of the before period—if significantly different from zero at the 5% level—has been removed from the dependent variable prior to the estimation. If location-based fixed effects are included in the regression, the parentheses show standard errors clustered at the level of the fixed effects variable. Otherwise, the parentheses show robust standard errors. The unconditional pre-treatment mean of the outcome variable and the removed pre-trend (if any) on a per-year basis are shown in the bottom panel.

Figure A1: Year fixed effects on direct PCI center admissions in different geographical subsamples before and after the budget cap exemption

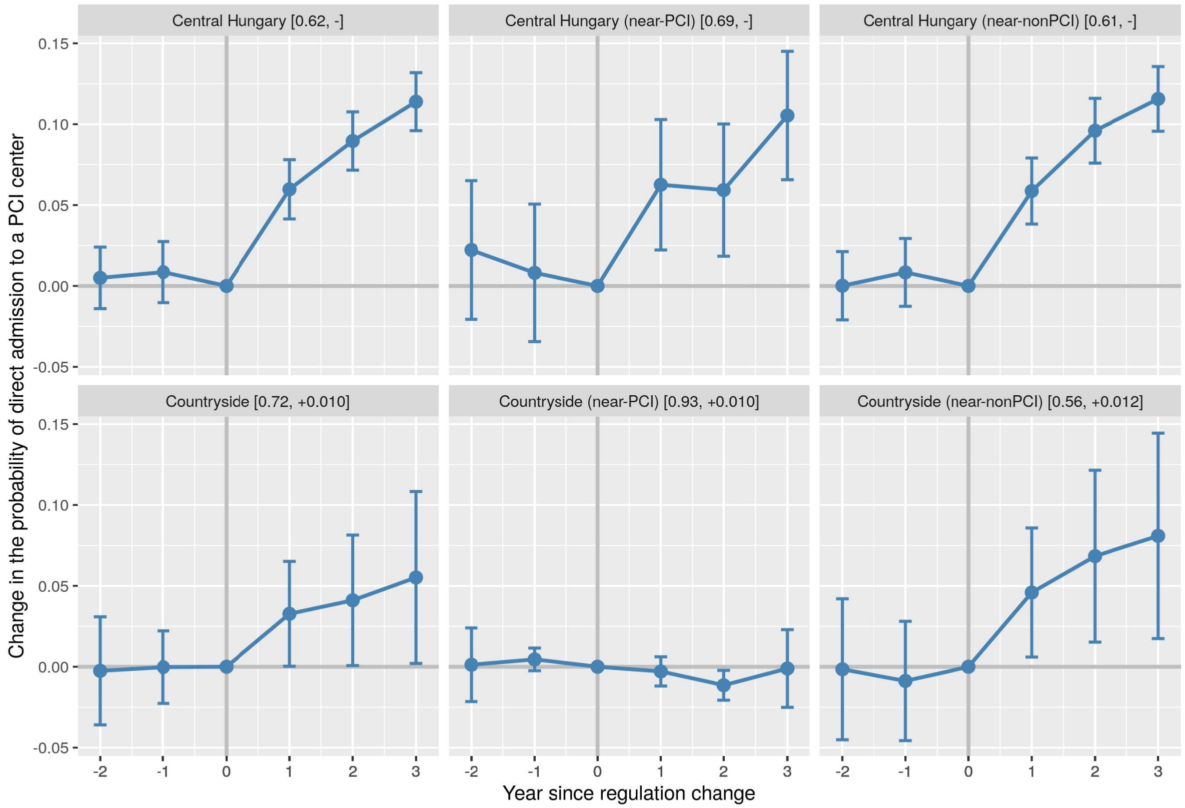

Note: The figure shows the estimated year fixed effects (β) and 95% confidence intervals on the outcome variable in different geographical samples according to equation (1). Pre-treatment years: -2, -1, 0 (reference). Post-treatment years: +1, +2, +3. The daily linear trend of the before period—if significantly different from zero at the 5% level—has been removed from the dependent variable prior to the estimation. The brackets in the subfigure headers show the sample-specific unconditional pre-treatment mean of the outcome variable, followed by the removed pre-trend (if any) on a per-year basis. See Table A4 for further details.

Figure A2: Year fixed effects on PCI center admissions by hospital transfer in different geographical subsamples before and after the budget cap exemption

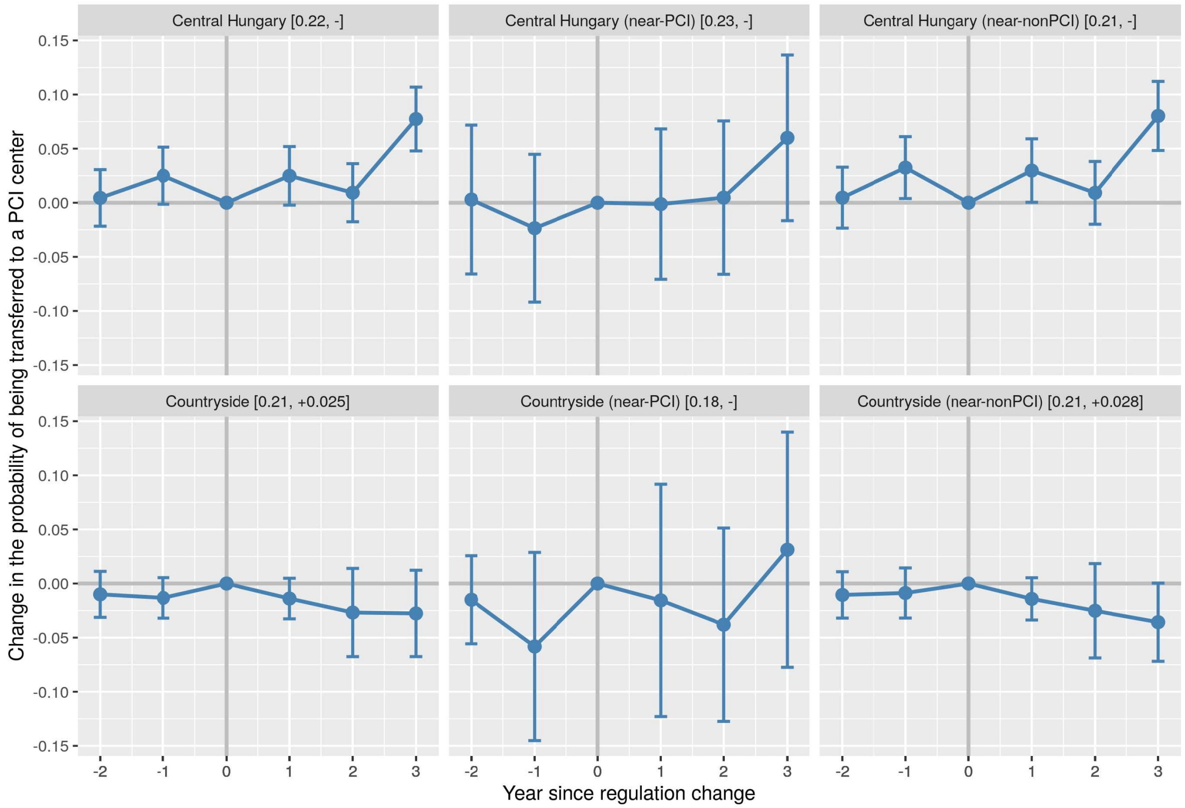

Note: The figure shows the estimated year fixed effects (β) and 95% confidence intervals on the outcome variable in different geographical samples according to equation (1). Pre-treatment years: -2, -1, 0 (reference). Post-treatment years: $+1$, $+2$, $+3$. The daily linear trend of the before period—if significantly different from zero at the 5% level—has been removed from the dependent variable prior to the estimation. The brackets in the subfigure headers show the sample-specific unconditional pre-treatment mean of the outcome variable, followed by the removed pre-trend (if any) on a per-year basis. See Table A5 for further details.

Figure A3: Year fixed effects on PCI treatment probability among directly or indirectly admitted PCI center patients in different geographical subsamples before and after the budget cap exemption

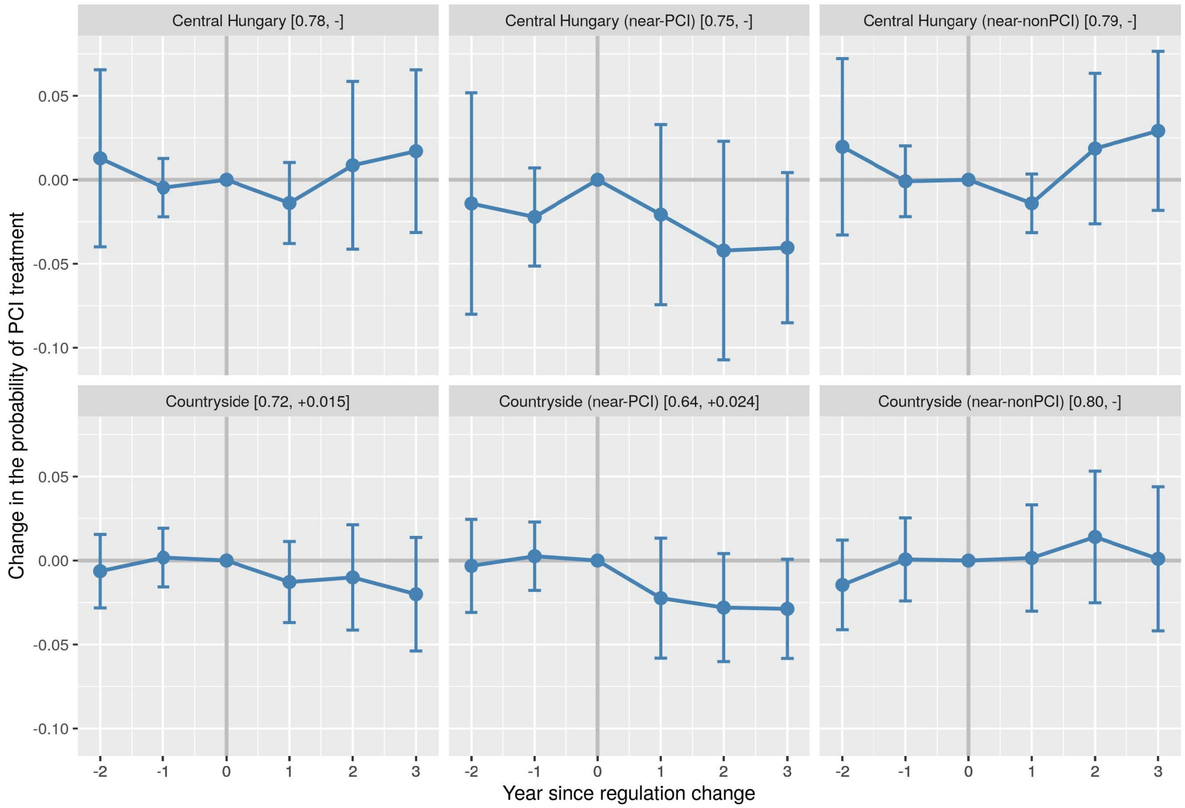

Note: The figure shows the estimated year fixed effects (β) and 95% confidence intervals on the outcome variable in different geographical samples according to equation (1). Pre-treatment years: -2, -1, 0 (reference). Post-treatment years: +1, +2, +3. The daily linear trend of the before period—if significantly different from zero at the 5% level—has been removed from the dependent variable prior to the estimation. The brackets in the subfigure headers show the sample-specific unconditional pre-treatment mean of the outcome variable, followed by the removed pre-trend (if any) on a per-year basis. See Table A6 for further details.

Figure A4: Year fixed effects on PCI treatment probability among directly admitted PCI center patients in different geographical subsamples before and after the budget cap exemption

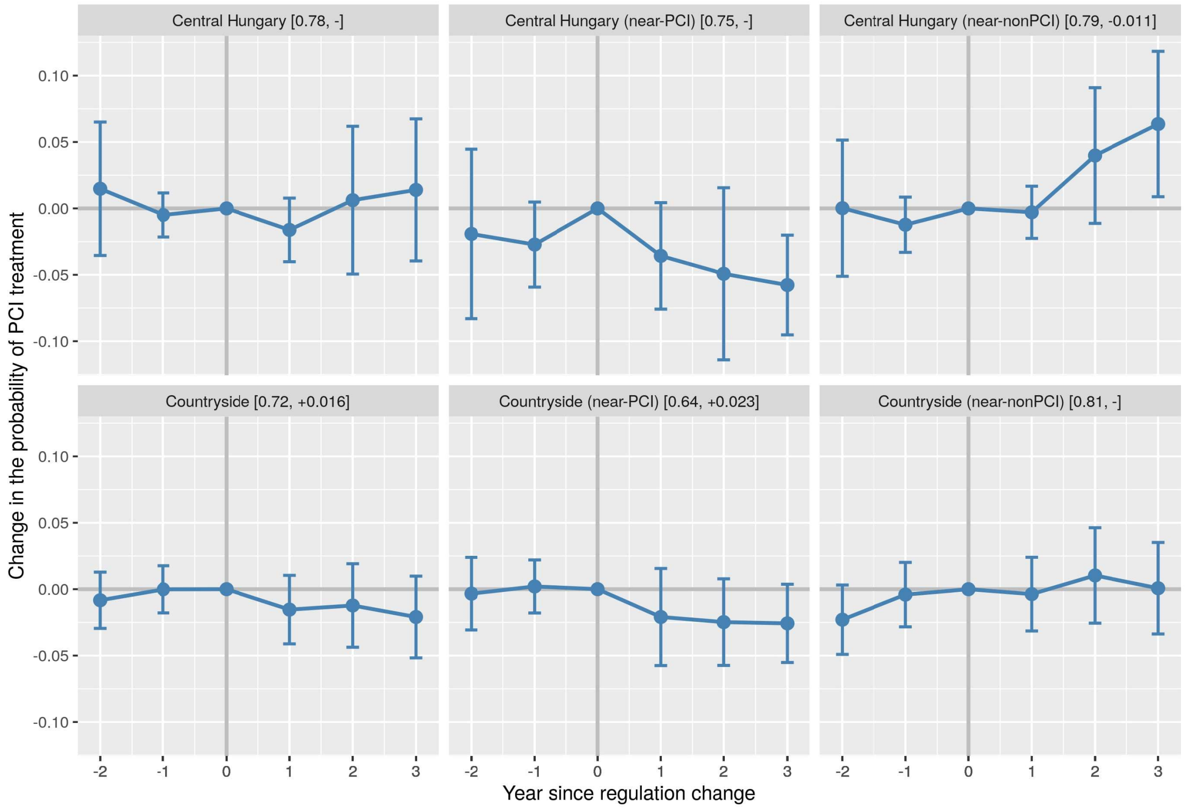

Note: The figure shows the estimated year fixed effects (β) and 95% confidence intervals on the outcome variable in different geographical samples according to equation (1). Pre-treatment years: -2, -1, 0 (reference). Post-treatment years: +1, +2, +3. The daily linear trend of the before period—if significantly different from zero at the 5% level—has been removed from the dependent variable prior to the estimation. The brackets in the subfigure headers show the sample-specific unconditional pre-treatment mean of the outcome variable, followed by the removed pre-trend (if any) on a per-year basis. See Table A7 for further details.

Figure A5: Year fixed effects on PCI treatment probability among PCI center patients admitted by hospital transfer in different geographical subsamples before and after the budget cap exemption

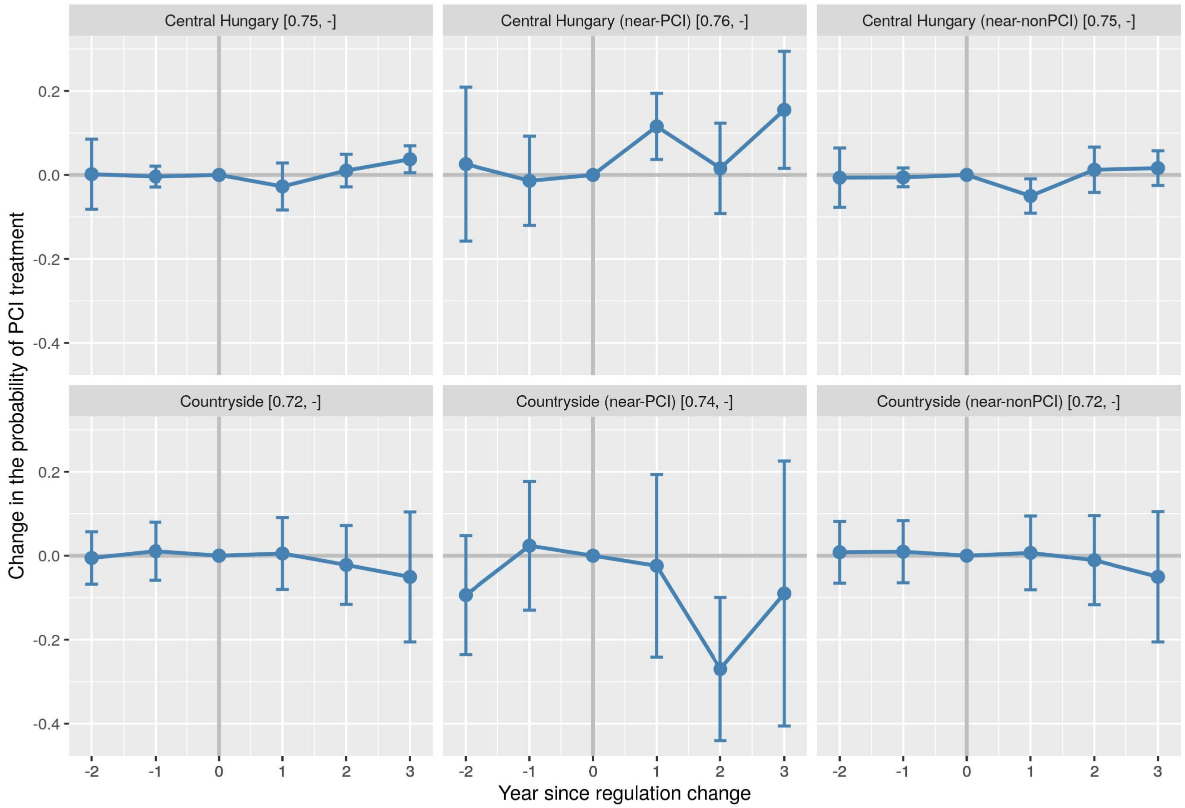

Note: The figure shows the estimated year fixed effects (β) and 95% confidence intervals on the outcome variable in different geographical samples according to equation (1). Pre-treatment years: -2, -1, 0 (reference). Post-treatment years: $+1$, $+2$, $+3$. The daily linear trend of the before period—if significantly different from zero at the 5% level—has been removed from the dependent variable prior to the estimation. The brackets in the subfigure headers show the sample-specific unconditional pre-treatment mean of the outcome variable, followed by the removed pre-trend (if any) on a per-year basis. See Table A8 for further details.

Figure A6: Year fixed effects on length of hospital stay (in days) in different geographical subsamples before and after the budget cap exemption

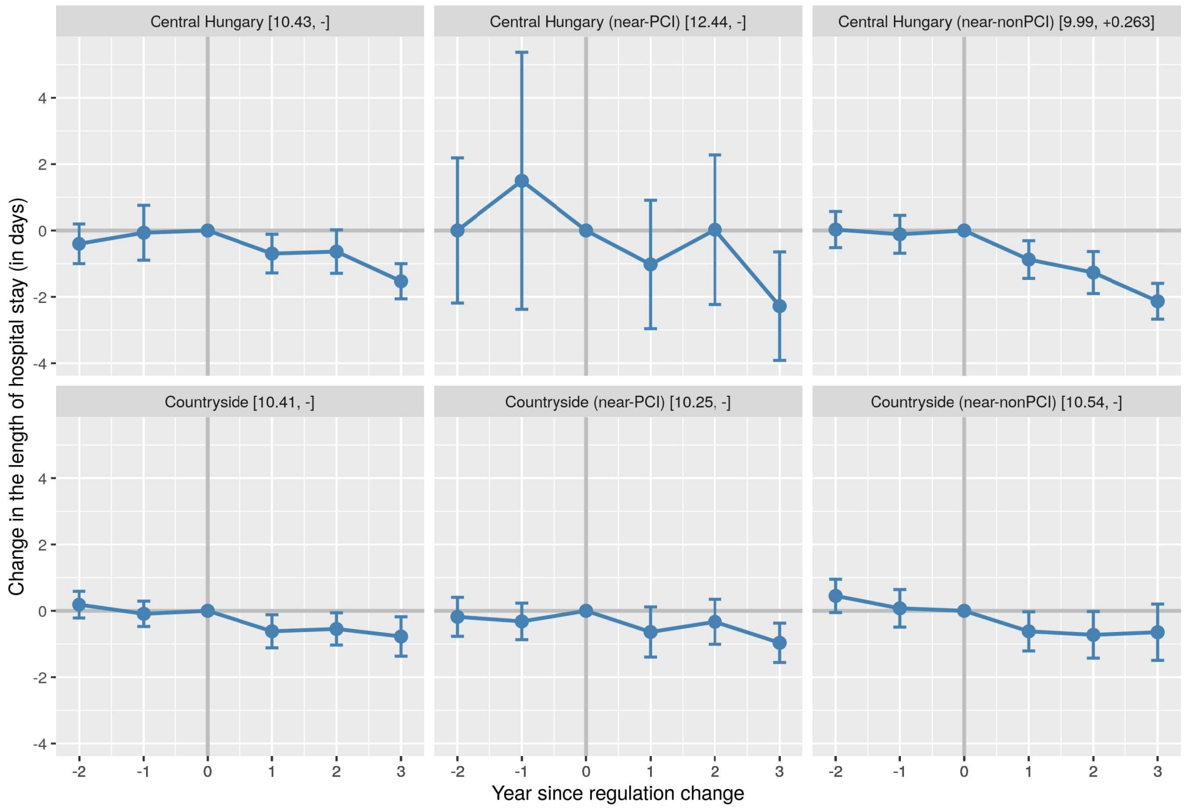

Note: The figure shows the estimated year fixed effects (β) and 95% confidence intervals on the outcome variable in different geographical samples according to equation (1). Pre-treatment years: -2, -1, 0 (reference). Post-treatment years: $+1$, $+2$, $+3$. The daily linear trend of the before period—if significantly different from zero at the 5% level—has been removed from the dependent variable prior to the estimation. The brackets in the subfigure headers show the sample-specific unconditional pre-treatment mean of the outcome variable, followed by the removed pre-trend (if any) on a per-year basis. See Table A9 for further details.

Figure A7: Year fixed effects on 30-day readmission probability with another AMI episode in different geographical subsamples before and after the budget cap exemption

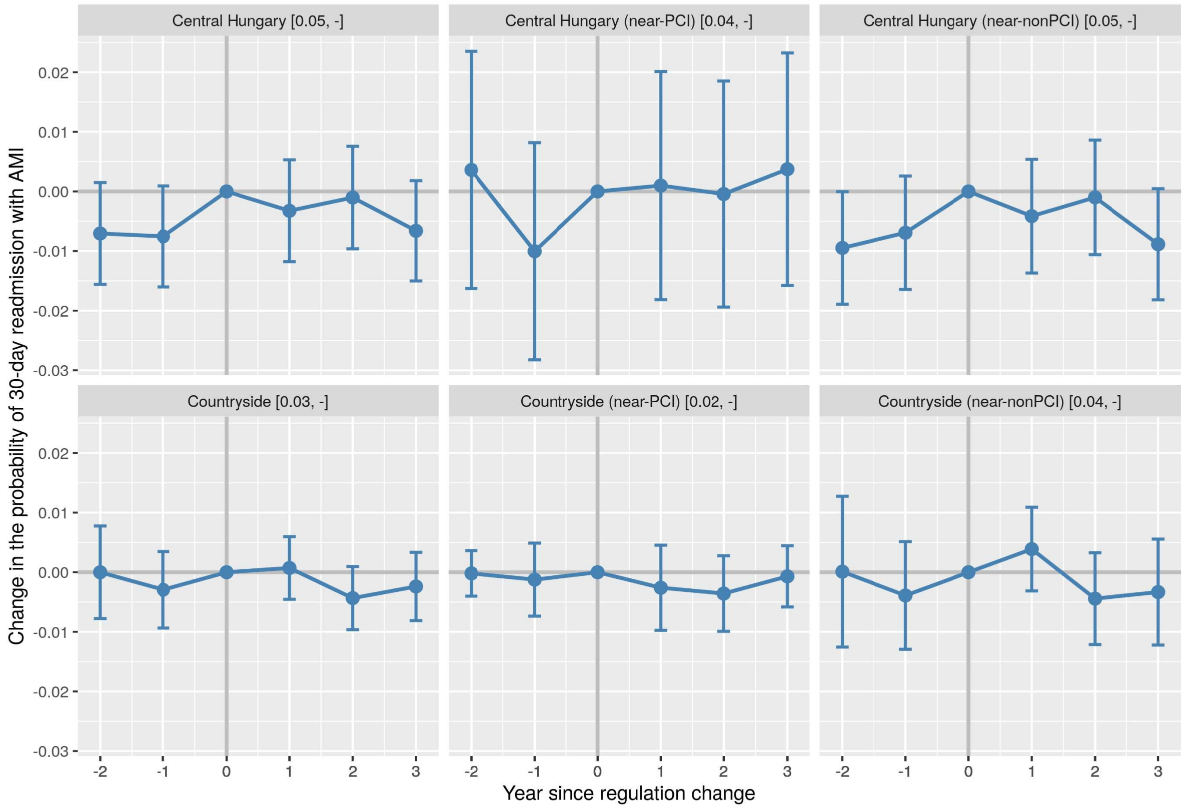

Note: The figure shows the estimated year fixed effects (β) and 95% confidence intervals on the outcome variable in different geographical samples according to equation (1). Pre-treatment years: -2, -1, 0 (reference). Post-treatment years: +1, +2, +3. The daily linear trend of the before period—if significantly different from zero at the 5% level—has been removed from the dependent variable prior to the estimation. The brackets in the subfigure headers show the sample-specific unconditional pre-treatment mean of the outcome variable, followed by the removed pre-trend (if any) on a per-year basis. See Table A10 for further details.

Figure A8: Year fixed effects on in-hospital mortality in different geographical subsamples before and after the budget cap exemption

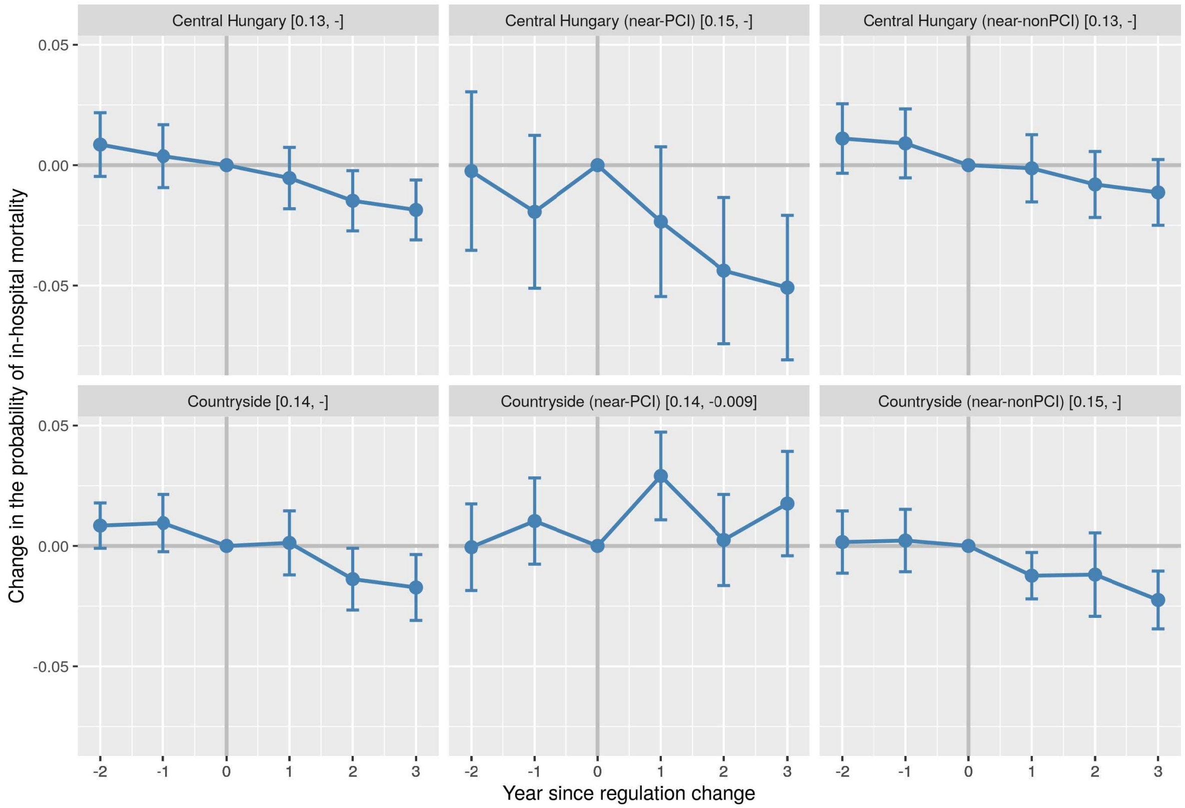

Note: The figure shows the estimated year fixed effects (β) and 95% confidence intervals on the outcome variable in different geographical samples according to equation (1). Pre-treatment years: -2, -1, 0 (reference). Post-treatment years: $+1$, $+2$, $+3$. The daily linear trend of the before period—if significantly different from zero at the 5% level—has been removed from the dependent variable prior to the estimation. The brackets in the subfigure headers show the sample-specific unconditional pre-treatment mean of the outcome variable, followed by the removed pre-trend (if any) on a per-year basis. See Table A11 for further details.
